# Profile of 6 microRNA in blood plasma distinguish early stage Alzheimer’s disease patients from non-demented subjects

**DOI:** 10.18632/oncotarget.15109

**Published:** 2017-02-05

**Authors:** Siranjeevi Nagaraj, Katarzyna Laskowska-Kaszub, Konrad J. Dębski, Joanna Wojsiat, Michał Dąbrowski, Tomasz Gabryelewicz, Jacek Kuźnicki, Urszula Wojda

**Affiliations:** ^1^ Laboratory of Preclinical Testing of Higher Standard, Nencki Institute of Experimental Biology of Polish Academy of Sciences, Warsaw, Poland; ^2^ Laboratory of Bioinformatics, Nencki Institute of Experimental Biology of Polish Academy of Sciences, Warsaw, Poland; ^3^ Department of Neurodegenerative Disorders, Mossakowski Medical Research Center, Warsaw, Poland; ^4^ Laboratory of Neurodegeneration, International Institute of Molecular and Cell Biology, Warsaw, Poland

**Keywords:** Alzheimer’s disease, early Alzheimers disease, mild cognitive impairment, microRNA, biomarker, Gerotarget

## Abstract

Alzheimers disease (AD) is the most common age-related dementia. Among its major challenges is identifying molecular signatures characteristic for the early AD stage in patients with Mild Cognitive Impairment (MCI-AD), which could serve for deciphering the AD pathomechanism and also as non-invasive, easy-to-access biomarkers. Using qRT-PCR we compared the microRNA (miRNA) profiles in blood plasma of 15 MCI-AD patients, whose diagnoses were confirmed by cerebrospinal fluid (CSF) biomarkers, with 20 AD patients and 15 non-demented, age-matched individuals (CTR).

To minimize methodological variability, we adhered to standardization of blood and CSF assays recommended by the international Joint Programming for Neurodegenerative Diseases (JPND) BIOMARKAPD consortium, and we employed commercially available Exiqon qRT-PCR-assays. In the first screening, we assessed 179 miRNAs of plasma. We confirmed 23 miRNAs reported earlier as AD biomarker candidates in blood and found 26 novel differential miRNAs between AD and control subjects. For representative 15 differential miRNAs, the TargetScan, MirTarBase and KEGG database analysis indicated putative protein targets among such AD hallmarks as MAPT (Tau), proteins involved in amyloidogenic proteolysis, and in apoptosis. These 15 miRNAs were verified in separate, subsequent subject groups. Finally, 6 miRNAs (3 not yet reported in AD context and 3 reported in AD blood) were selected as the most promising biomarker candidates differentiating early AD from controls with the highest fold changes (from 1.32 to 14.72), consistent significance, specificities from 0.78 to 1 and sensitivities from 0.75 to 1. (patent pending, PCT/IB2016/052440).

## INTRODUCTION

The most common cause of age-related dementia worldwide is Alzheimer’s disease (AD), a neurodegenerative disorder that affects 47 million individuals globally and is foreseen to increase to about 76 million in 2030 [1]. The global costs related to AD are similar to the financial burden of heart disease and cancer, placing AD among the major unmet health concerns [2]. Although mounting evidence indicates that for overcoming AD critically important is its early detection, before massive neuron loss occurs, readily available tests that can give an early and reliable AD diagnosis are lacking.

Clinical AD symptoms develop after a preclinical, asymptomatic pathogenic process several decades long [3]. The early clinical AD stage occurs not in all, but in some patients with Mild Cognitive Impairment (MCI) [4, 5]. AD progresses from early and mild, through moderate, to the severe, late stage. The recently updated clinical diagnostic criteria allow only for the probable or possible diagnosis of AD in patients with clinical dementia or MCI (MCI attributable to AD; early AD) [6–8]. A definite AD diagnosis is presently possible only based on the post mortem histopathological examination of the brain.

In current clinical practice, the diagnosis of AD starts with a clinical examination and neuropsychological testing such as the Mini-Mental State Examination (MMSE) and can be supported by a CSF biochemical assay of the levels of “total” and hyperphosphorylated tau protein, and the 42-amino-acid Aβ peptide, that reflect brain pathology. Brain imaging has evolved to be as efficient as CSF analysis, with an even higher earlier-diagnosis capacity [8]. However, the high costs and requirements for sophisticated equipment represent a fundamental barrier for the application of brain imaging as a large-scale AD screening tool in most clinical settings. In turn, although CSF seems to reflect the biochemical changes occurring in the brain, CSF assays require a lumbar puncture which is an invasive procedure, not always feasible, especially in the elderly. Because of limits involved in frequent, multiple lumbar punctures, CSF assays are also not suitable for the concurrent monitoring of therapeutic trials, drug efficacy, and for longitudinal studies. Therefore, while the recent development of technologies addressing the ‘AD signature’ in CSF reflects significant progress in AD diagnosis, CSF biomarkers are not likely to fulfill optimal diagnostic criteria for clinical practice.

As indicated by the Food and Drug Administration (FDA), a preferable biomarker for clinical applications should be available in biological samples that are easy to obtain in a safe, non-invasive procedure, and the laboratory methods must be reliable, stable, and cost-effective. In this light, progress in AD diagnostics relies to a great extent on the identification of novel AD biomarkers in more easily available diagnostic tissues, such as blood [8, 9]. Accordingly, all systemic paradigms have been recently explored in the search for blood-based AD molecular signatures, including proteomics, lipidomics, transcriptomics, metabolomics, and epigenomics (reviewed in [9]).

One of the most promising approaches to the identification of blood-based AD biomarkers concentrates on circulating miRNAs [9–11]. Possible application of miRNAs as AD biomarkers was prompted by increasing evidence of the significant regulatory functions of miRNA in different pathologies including neurodegeneration, and the altered expression of miRNAs reported in many disease states in biofluids [12–14]. miRNAs are abundant in the blood and can operate both in adjacent cells as well as in more distant areas of the body *via* a mechanism similar to hormones [13–15]. miRNAs show an advantage as a biomarkers, being exceptionally stable among blood macromolecules, as they have been reported to be transported in blood in exosomes, high-density lipoproteins, and in complexes with proteins, protecting them from degradation [16–19].

For AD, so far 31 investigations indicated potential for miRNAs as biomarkers in the blood (serum, plasma, blood cells, and whole blood were analyzed) [20–46, 84–87]. However, broader reproducibility of the results has not yet been achieved, which is currently seen as, to a high degree, the result of substantial variability across various diagnostic centers in pre-analytical, analytical and post-analytical factors, referring to both the diagnosis of patients recruited for studies, and to the methodology of blood handling and miRNA analysis [47, 48]. Moreover, so far in patients with MCI due to AD only one study reported miRNA signatures that could be directly related not only to neuropsychological tests, but also the levels of CSF biomarkers [87]. In previous studies of miRNA AD signatures in blood, CSF markers assessment was mostly absent [20–22, 24–28] or available only for AD patients [23]. The aim of this study was to partly respond to these needs.

Our study involved MCI patients with a likelihood that the MCI was due to AD, as confirmed by the levels of CSF markers (patients denoted as MCI-AD). In the preliminary and verification steps of the study altogether we compared 15 early AD patients (MCI-AD) with 20 later AD patients (AD) and with 15 non-demented, age-matched control subjects. All involved patients were diagnosed in adherence to the current golden standards [6, 7]. To respond to the necessity for the extensive standardization of materials and procedures, we employed standardized CSF assays and guidelines for the standardization of pre-analytic variables for blood-based biomarkers established under the international JPND BIOMARKAPD consortium [47]. To further standardize our research, we applied a commercially available qRT-PCR assay comprising 179 miRNAs preselected for blood plasma. Based on the pilot screening using this assay, we selected 15 miRNAs, consisting of 6 miRNAs previously reported as AD biomarker candidates and 9 novel candidates for further validation. Finally, 6 miRNAs (3 novel and 3 reported earlier) were selected as the most promising early AD biomarker candidates (6miR) for future verification in larger cohorts. The results suggest that miRNA blood biomarkers can be further tested as a possible replacement for CSF markers as identifiers of early AD.

## RESULTS

**Figure 1:**
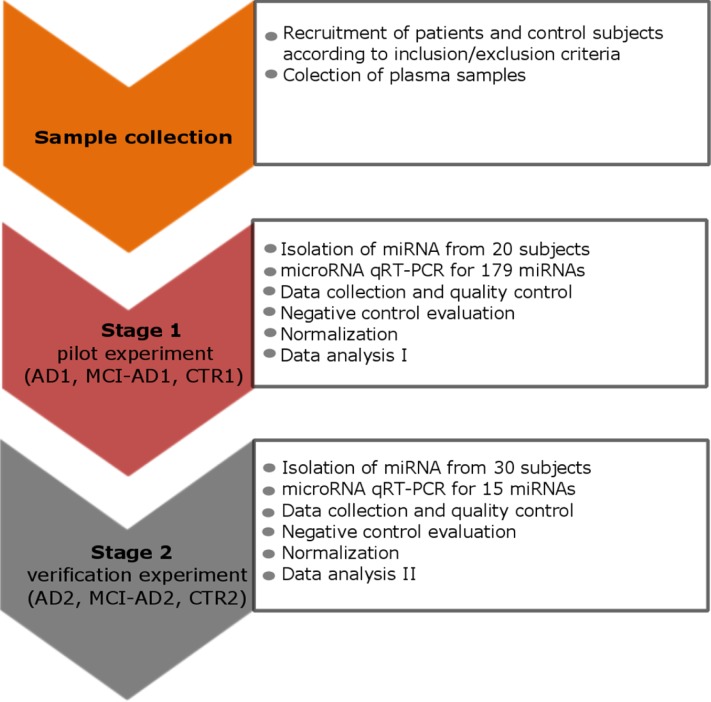
The study design scheme that outlines the two stages: the pilot and the verification stage Stage 1 and Stage 2 involved different, independent groups of subjects: in the pilot stage 1 control subjects CTR1, AD1 patients and MCI-AD1 patients were enrolled; in the verification stage 2 control subjects CTR2, AD2 patients and MCI-AD2 patients were enrolled. Patients’ diagnostic data are described in Table 1.

We compared plasma miRNA profiles of early AD patients with more advanced AD patients (moderate AD), whose diagnoses were supported by CSF markers, and with two separate, non-demented, age-matched control groups. The study design is schematically shown in Figure 1. The study consisted of two stages of profiling miRNA in blood plasma samples: the pilot experiment (Stage 1) and the verification experiment (Stage 2). Patient groups enrolled adequately in Stage 1 and Stage 2 and patients’ diagnostic data are described in Table 1.

**Table 1:** Patients characteristics in all groups enrolled in the study

Group	Size	Age	Sex (f/m)	MMSE	CDR	Aβ42 (pg/ml)	t-tau (pg/ml)	p-tau (pg/ml)
AD 1	7	73.7±5	3/4	19.85±4	1	380.4±115	799.6±268	95.1±24
AD 2	13	67.5±8	7/6	20.92±4	1.4	394.4±188	749±392	94.5±45
MCI-AD 1	7	64.3±6	3/4	26.14±2	0.5	700.4±443	355.1±67	55.3±13
MCI-AD 2	8	65.8±7	5/3	25.62±3	0.5	563.4±262	532.9±312	79.3±37
**AD_ALL_**	**20**	**67±8**	**10/10**	**20.4±4**	**1.2**	**387.4±163**	**774.3±347**	**94.8±38**
**MCI-AD_ALL_**	**15**	**62±6**	**8/7**	**25.9±2**	**0.5**	**631.9±351**	**444±243**	**67.3±30**

AD, patients with developed Alzheimer’s disease; MCI-AD, mild cognitive impairment patients with probable early AD. Patients enrolled in preliminary experiments (stage 1): AD1, MCI-AD1. Patients enrolled in verification experiments (stage 2): AD2, MCI-AD2. All patients in summary enrolled in both stage 1 and stage 2: AD_all_, MCI-AD_all_. MMSE- Mini-Mental State Examination, CDR- Clinical Dementia Rating. CSF tests results: Aβ42, amyloid beta 42; t-tau, total tau; p-tau, phosphorylated tau; mean values +/-SD are shown. All patients showed AD-type neuronal injuries in the hippocampus in their MRIs.

### Stage 1: pilot screening for miRNA biomarkers

The pilot experiment was performed in the following three groups of subjects: in 7 patients diagnosed with clinical AD (AD1), in 7 patients diagnosed with MCI due to AD (early AD, MCI-AD1) and in 6 healthy non-demented age-matched individuals (CTR1); (Table 1, Table 2). In the pilot experiment, we used qRT-PCR-based Exiqon panel which allows for the assessment of 179 miRNA levels in blood plasma (miRCURY LNA focus panel). These 179 miRNAs in the focus panel were carefully selected based on over 1 million data points from human blood serum/plasma samples collected from healthy as well as diseased individuals available at Exiqon and at collaborative sources. For the selection of the relevant miRNAs for this panel, miRNA expression data from various types of cancer, neurological disorders/neurodegenerative diseases and inflammation/allergies were used. Importantly, Exiqon’s database is largely based on total RNA extracted from human blood serum or plasma, meaning that a particular circulating miRNA can be detected by the applied focus panel, irrespective of whether it is preferentially protein bound, found in exosomes, micro-vesicles or otherwise compartmentalized, resulting in a panel allowing for a very comprehensive blood plasma/serum miRNA assay.

**Table 2:** Characteristics of subject groups enrolled in the Stage 1 (pilot) experiment

Sample group	Cohort size	Age (mean +/- SD)	Sex (female/male)
**AD1**	7	73.7 +/-5	3/4
**MCI-AD1** (early AD)	7	64.3 +/-6	3/4
**CTR1** (non-demented)	6	66 +/-5	4/2

AD1, patients with developed Alzheimer’s disease; MCI-AD1, MCI patients with probable early AD; CTR1, control non-demented age-matched individuals.

All samples of miRNA detection from each subject were analyzed in triplicate by standardized Exiqon methodology on miRCURY LNA focus panel by qRT-PCR using Roche Lightcycler 480. All analyzed plasma samples showed no contamination by blood cells based on the hemolysis assay. The analysis of our pilot study results showed that an average of 170 miRNAs out of 179 were detected per sample. The minimal number of identified miRNAs in a sample was 153. For normalization in Stage 1, we used all samples from all subject groups (*n* = 20): AD1 (*n* = 7), MCI-AD1 (*n* = 7) and CTR1 (*n* = 6). 153 out of 179 assays were detected in all subject groups. Average Cq of 153 assays from these 20 samples were used for normalization.

We started searching for miRNAs which could differentiate early AD patients from non-demented subjects by first identifying miRNAs which differentiated patients with fully developed AD (AD1) from control subjects and we indeed found 32 of such miRNAs using *t*-test *p*-value cutoff < 0.05 (Table 3). Stringent analysis using one-way ANOVA showed differences between the AD1, MCI-AD1 and CTR1 groups: ANOVA *p* value < 0.05; FDR < 0.25 (Supplementary Figure 1). One-way ANOVA with post-hoc Tukey test HSD (honestly significant difference) *p*-value for AD1 *vs* CTR1 indicated 13 miRNAs with *p* < 0.05 out of the 32 shown in Table 3, and these miRNAs are underlined in Table 3. Out of 13 miRNAs, we found 6 miRNAs with *p* < 0.01 and 7 miRNAs with *p* < 0.05. By accepting higher stringency of *p* values as selection criteria for differential miRNAs we could better limit false positives. However, in the pilot screening it is also important not to lose too many potential differential miRNAs which could be further verified and which could show some biological significance. Therefore we based our selection criteria for candidate miRNAs to be further tested in the verification step on statistical cutoff value of *p* < 0.05, and also on fold change differences over 1.2 and concordant directionality comparing AD1 *vs* CTR1 and MCI-AD1 *vs* CTR1 groups. Thus, we selected all the 13 miRNAs (underlined and marked bold in Table 3) for the verification experiment in the next groups of AD2, MCI-AD2 and CTR2 subjects. We also selected for verification 2 more miRNAs (has-miR-486-5p, has-miR-151a-5p) which showed fold change differences over 1.2 and concordant directionality in both AD1 *vs* CTR1 and MCI-AD1 *vs* CTR1 (marked bold in Table 3).

**Table 3:** miRNAs found using a *t*-test *p*-value cutoff < 0.05 to be differentially expressed between the group of AD patients (AD1) and non-demented age-matched controls (control group 1, CTR1)

Comparison	*p*-value	Differentially expressed miRNAs
AD1 *vs* CTR1	*p < 0.05	hsa-let-7d-3p, hsa-miR-423-5p, **hsa-miR-30b-5p, hsa-miR-200a-3p, hsa-miR-142-3p**, hsa-let-7g-5p, **hsa-miR-320c, hsa-miR-320a,** hsa-miR-2110, **hsa-miR-320b**, hsa-miR-15b-3p, hsa-let-7f-5p, hsa-let-7b-5p, **hsa-miR-18a-5p**, hsa-miR-10b-5p, **hsa-miR-301a-3p, hsa-miR-1260a, hsa-miR-502-3p**, hsa-miR-374a-5p, **hsa-miR-483-5p**, hsa-let-7d-5p, **hsa-miR-103a-3p**, hsa-miR-574-3p, **hsa-miR-33a-5p**, hsa-miR-320d, hsa-miR-22-5p, hsa-miR-374b-5p, hsa-miR-193a-5p, **hsa-miR-151a-5p, hsa-miR-486-5p**, hsa-miR-186-5p, hsa-miR-30c-5p

Underlined bold miRNAs were also found significant in AD1 *vs* CTR1 using one-way ANOVA with post-hoc Tukey HSD (honestly significant difference), *p*-value for multiple comparisons. miRNAs chosen for further verification in stage 2 are marked in bold.

**Figure 2:**
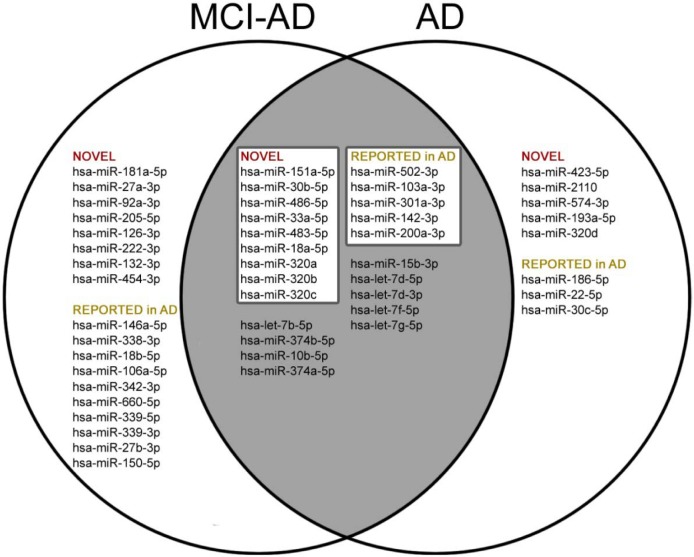
Comparison of deregulated miRNAs (*t*-test, *p* < 0.05) reported so far in 31 publications [20–46, 84–87] in AD blood (whole blood, blood cells, plasma, serum) found by literature search in Web of Science (Collection of Databases) with terms “MicroRNA” or “miRNA” AND “Blood” AND “Alzheimer disease”, with data of our pilot study (*t*-test, *p* < 0.05)

Next we compared results of our pilot study with the data from all 31 previous reports on miRNAs in AD blood (whole blood, blood cells, serum or plasma), obtained using different methodology (Figure 2). All 31 previously published articles reported altogether 122 differential miRNAs in blood as potential AD biomarkers. 73 miRNAs out of the 122 so far reported were present in the 179 miRNA panel used in our pilot study. Out of these 73 miRNAs, 23 miRNAs were reproduced as significantly differential in AD1 and/or MCI-AD1 patients compared to controls CTR1 (*t*-test *p* < 0.05) (Figure 2). Overall, in our study 50 miRNAs were found to be significantly deregulated in AD1 and MCI-AD1 *versus* CTR1: 26 novel miRNAs, not reported previously as potential AD blood biomarkers, and 24 miRNAs which were previously reported as differential in AD: 23 miRNAs reported in AD blood (Figure 2) and 1 miRNA reported earlier as differential in AD brain (hsa-miR-1260a [57]). Among novel and reported miRNAs differentiating patient samples from controls, some were present only in AD1 or only in MCI-AD1 patients, whereas 23 miRNAs, including 15 miRNAs selected for stage 2 verification, were common for AD1 and MCI-AD1 (Figure 2).

**Figure 3:**
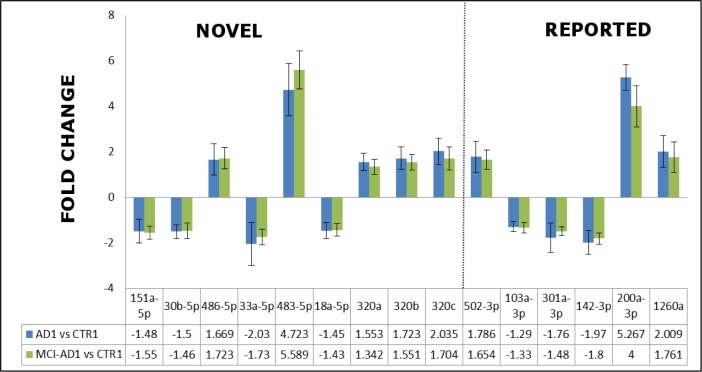
The fold changes in the expression levels determined by qRT-PCR in the pilot experiment/Stage 1 for the representative 15-miRNAs differentiating AD patients AD1 *versus* non-demented controls CTR1, and early AD patients MCI-AD1 *versus* non-demented controls CTR1 9 miRNAs reported for the first time as AD biomarker candidates are indicated as separate field of the plot. Means + SD are shown from triplicate experiments.

Among these 15 selected miRNAs, 9 miRNAs were newly identified, not reported previously as potential blood biomarkers of AD: hsa-miR-151a-5p, hsa-miR-30b-5p, hsa-miR-486-5p, hsa-miR-33a-5p, hsa-miR-483-5p, hsa-miR-18a-5p, hsa-miR-320a, hsa-miR-320b, hsa-miR-320c (Figure 2, Figure 3). Furthermore, the remaining selected miRNAs were previously reported ones: 5 miRNAs which were previously reported in 5 different studies as potential AD blood biomarkers (hsa-miR-502-3p [28], hsa-miR-103a-3p [22], hsa-miR-301a-3p [21], hsa-miR-142-3p [21, 87], and miR-200a-3p [30]); and also 1 differential miRNA which was earlier reported in AD brain (hsa-miR-1260a [57]). The 15 miRNAs, including 9 novel and those previously reported in AD context, were next verified as potential differential plasma miRNAs for earlier (and later) AD compared to controls. Sequences for all 15 selected miRNAs are shown in Supplementary Table 1.

### Stage 2: verification experiment of miRNA biomarkers in the next groups of subjects

In the second experimental stage, we used the same methodology for validating the pilot experiment results through the analysis of the levels of the 15 differential miRNAs selected in the first stage. For normalization, additional 5 miRNAs were assayed, which proved to be most stable in the pilot screening (hsa-miR-185-5p, hsa-miR-128-3p, hsa-miR-130b-3p, hsa-miR-15a-5p, hsa-miR-425-3p). The levels of these 20 miRNAs have been analyzed in three novel, separate groups of subjects (Table 1, Table 4).

**Table 4:** Characteristics of subject groups enrolled in the Stage 2 (verification) experiment

Sample group	Cohort size	Age (mean +/- SD)	Sex (female/male)
**AD2**	13	67.5 +/- 8	7/6
**MCI-AD2** (early AD)	8	65.8 +/- 7	5/3
**CTR2** (non-demented)	9	66 +/- 3	4/5

AD2, second group of patients with developed Alzheimer’s disease; MCI-AD2, second group of MCI patients with probable early AD; CTR2, second control group of non-demented age-matched individuals.

**Figure 4:**
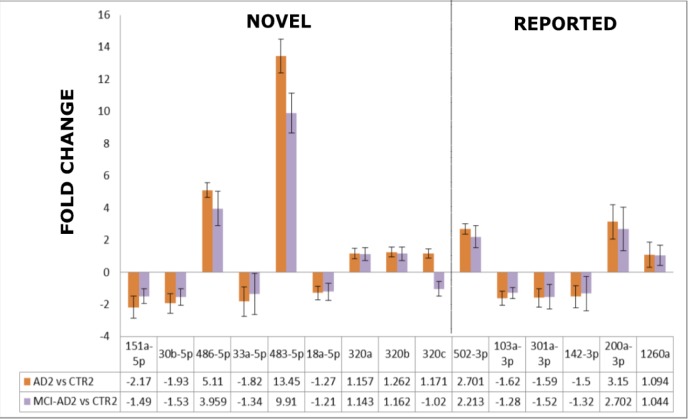
The comparison of the fold changes in the expression levels obtained by qRT-PCR in the verification experiment/Stage 2 for the 15-miRNAs which differentiated AD2 and MCI-AD2 patients *versus* non-demented controls CTR2 9 miRNAs reported for the first time as AD biomarker candidates are indicated as a separate field of the plot. Means + SD are shown from triplicate experiments.

As shown in Figure 4 and Table 5, this independent experiment of Stage 2 confirmed in overall the direction of deregulation of the 15 selected miRNAs in AD blood plasma, including the 9 novel miRNA biomarkers identified in our pilot screening. When we compared the fold change and direction of deregulation for all 15 miRNAs in the verification and pilot experiments (Figure 4. Figure 3), the results proved to be very consistent. Thus it should be stressed that in all AD patient groups (AD1, AD2, MCI-AD1, MCI-AD2; 35 patients) compared with two different control groups (CTR1 and CTR2, 15 subjects), data for all 15 miRNAs of the tested miRNAs, including the 9 novel miRNAs, are overall concordant (except for hsa-miR-320c) in terms of the direction of changes in miRNA levels (upregulation and downregulation) and in fold changes (Figure 4, Figure 3, Table 5). In addition, direction of changes for the control 4 out of 5 miRNAs previously reported in AD blood context proved to be concordant with the published results (Table 5).

**Table 5:** Comparison of directions of changes (↓decrease, ↑increase) in the plasma levels and statistical significance for the 15 miRNAs in the pilot and in the verification experiment

miRNA (hsa-miR)	AD1 *vs* CTR1	AD2 *vs* CTR2	MCI-AD1 *vs* CTR1	MCI-AD2 *vs* CTR2	Published earlier in AD	Ref.
151a-5p	↓	↓***	↓*	↓		
30b-5p	↓**	↓**	↓**	↓		
486-5p	↑	↑***	↑	↑***		
33a-5p	↓*	↓	↓	↑		
483-5p	↑*	↑****	↑**	↑***		
18a-5p	↓*	↓	↓*	↓		
320a	↑**	↑	↑	↑		
320b	↑**	↑	↑*	↑		
320c	↑**	↑	↑*	↓		
502-3p	↑*	↑****	↑	↑**	Whole blood (↓)	[28]
103a-3p	↓*	↓**	↓*	↓	Whole blood (↓)	[22]
301a-3p	↓*	↓	↓	↓	Plasma (↓)	[21]
142-3p	↓**	↓	↓**	↓	Plasma (↓)	[21, 87]
200a-3p	↑**	↑**	↑*	↑*	Blood mononuclear cells (200a) (↑)	[30]
1260a	↑*	↑	↑	↑	Cortex (Brain)	[57]

For 6 miRNAs direction of changes for AD *versus* control were compared also with the published results. Data for 9 miRNAs reported here for the first time as AD biomarker candidates have no references. Statistical significance (one-way ANOVA with post-hoc Tukey HSD *p*-value): *p < 0.05, **p < 0.01, ***p < 0.001; ****p < 0.0001. Comparisons for AD patient groups *versus* control groups and for MCI-AD (early AD) patient groups *versus* control groups are shown.

Among the miRNAs which significantly differentiated MCI-AD patients from controls both in pilot and verification studies, two of the 9 novel miRNAs showed the highest fold changes (in the range of 4-13), and the highest statistical significance: hsa-miR-483-5p and hsa-miR-486-5p (Figure 4, Table 6). In particular, in the verification experiment which was performed in larger subject groups, the fold change in case of hsa-miR-483-5p was extremely high, in the range of 8-13 (Figure 4). Thus, these 2 miRNAs identified in our study: hsa-miR-486-5p, and particularly hsa-miR-483-5p, are the most significant indicators of early (and later) AD.

**Table 6:** Receiver Operating Characteristics (ROC) curve parameters for the 9 novel AD miRNA biomarker candidates and 6 previously reported miRNAs that separate all early and later stage AD patients versus all non-demented controls: AD *vs* CTR (AD1 and AD2 *vs* CTR1 and CTR2) and MCI-AD *vs* CTR (MCI-AD1 and MCI-AD2 *vs* CTR1 and CTR2)

		Train set (Pilot)			Test set (Verification)		
miRNAs (hsa-miR)	Comparison	Auc	SP	SN	Auc	SP	SN
**9 novel miRNA biomarker candidates**							
**151a-5p**	AD *vs* CTR	**0.83**	1	0.57	**0.90**	0.77	1
**151a-5p**	MCI-AD *vs* CTR	**0.93**	1	0.71	**0.79**	0.88	0.87
**30b-5p**	AD *vs* CTR	**0.95**	1	0.86	**0.88**	0.88	0.84
**30b-5p**	MCI-AD *vs* CTR	**0.95**	0.83	1	**0.82**	0.89	0.87
**486-5p**	AD *vs* CTR	**0.78**	1	0.57	**0.93**	0.89	1
**486-5p**	MCI-AD *vs* CTR	**0.90**	1	0.86	**0.87**	0.89	0.87
**33a-5p**	AD *vs* CTR	**0.86**	1	0.86	**0.78**	0.78	0.85
**33a-5p**	MCI-AD *vs* CTR	**1**	1	1	**0.80**	0.78	0.87
**483-5p**	AD *vs* CTR	**0.92**	1	0.8	**0.99**	1	0.92
**483-5p**	MCI-AD *vs* CTR	**0.93**	1	0.83	**0.95**	1	0.87
**18a-5p**	AD *vs* CTR	**0.90**	1	0.71	**0.79**	0.67	0.92
**18a-5p**	MCI-AD *vs* CTR	**0.95**	0.83	1	**0.73**	0.67	0.87
**320a**	AD *vs* CTR	**0.97**	1	0.86	**0.65**	0.67	0.69
**320a**	MCI-AD *vs* CTR	**0.93**	1	0.86	**0.76**	1	0.62
**320b**	AD *vs* CTR	**0.93**	1	0.86	**0.83**	1	0.61
**320b**	MCI-AD *vs* CTR	**0.95**	1	0.86	**0.79**	1	0.62
**320c**	AD *vs* CTR	**0.95**	0.83	1	**0.67**	1	0.38
**320c**	MCI-AD *vs* CTR	**0.93**	0.83	1	**0.58**	0.78	0.5
**6 reported miRNA biomarker candidates**							
**502-3p**	AD *vs* CTR	**0.90**	0.83	1	**0.94**	0.89	1
**502-3p**	MCI-AD *vs* CTR	**0.90**	0.83	0.86	**0.86**	0.89	0.87
**103a-3p**	AD *vs* CTR	**0.86**	0.83	0.86	**0.88**	0.89	0.85
**103a-3p**	MCI-AD *vs* CTR	**0.88**	0.83	0.86	**0.76**	0.89	0.75
**301a-3p**	AD *vs* CTR	**0.86**	1	0.71	**0.83**	0.78	0.92
**301a-3p**	MCI-AD *vs* CTR	**0.97**	1	0.86	**0.80**	0.78	0.87
**142-3p**	AD *vs* CTR	**0.95**	1	0.86	**0.79**	0.89	0.77
**142-3p**	MCI-AD *vs* CTR	**1**	1	1	**0.80**	0.78	0.87
**200a-3p**	AD *vs* CTR	**1**	1	1	**0.90**	1	0.77
**200a-3p**	MCI-AD *vs* CTR	**1**	1	1	**0.83**	0.89	0.75
**1260a**	AD *vs* CTR	**0.88**	1	0.71	**0.65**	0.78	0.69
**1260a**	MCI-AD *vs* CTR	**0.81**	1	0.57	**0.71**	0.66	0.87

Analysis was performed with two separate groups (pilot experiment/stage 1 and verification experiment/stage2). ROC curve analysis: Auc- area under the ROC curve, SP- Specificity, SN-Sensitivity.

In addition, a consistent and statistically significant upregulation of the previously reported hsa-miR-502-3p and hsa-miR-200a-3p has been observed in both the pilot and verification stage of our study, with approximately two-fold increased levels of these miRNAs in MCI-AD and AD samples compared to non-demented controls (Figure 3, Figure 4, Table 5).

### ROC analysis for pilot and verification study

**Figure 5:**
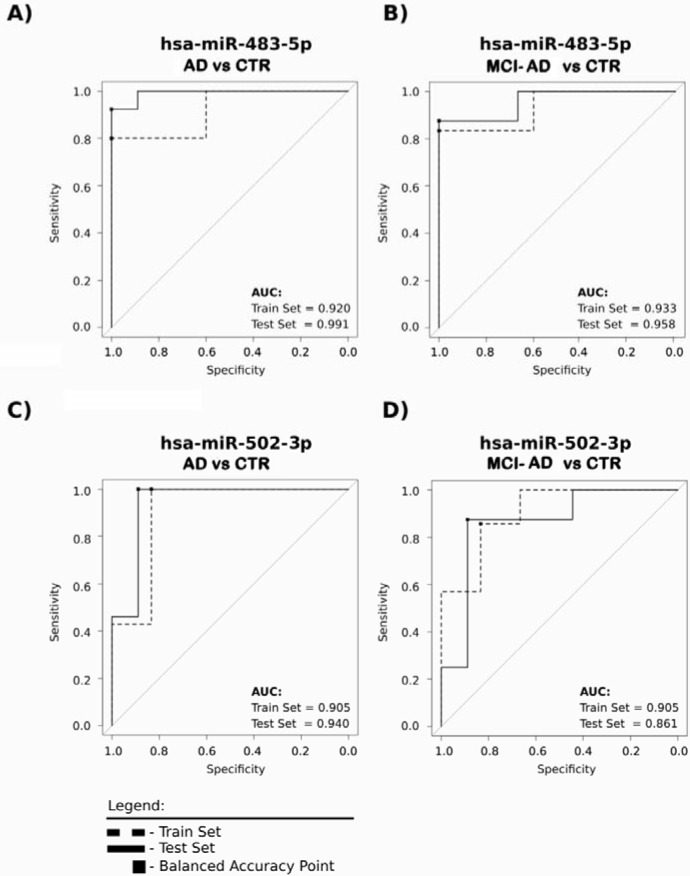
The two exampled miRNAs: hsa-miR-483-5p and hsa-miR-502-3p clearly distinguish with a high sensitivity and specificity early AD as well as AD patients from non-demented controls The receiver operating characteristic (ROC) curves were prepared for two groups, in which specificity and sensitivity were calculated for the balanced accuracy point (optimal operating threshold). Dashed line, data of the pilot experiment/Stage 1; solid line, data from verification experiment/Stage 2 to confirm the ability of each miRNA to properly separate the samples. ROC curve parameters for all analyzed 15 miRNAs are shown in Table 6.

Further, we tested the suitability of each of the 15 miRNAs of the verification study as potential blood-based AD biomarker candidates, by calculating the parameters in ROC curves. This analysis demonstrated that the 15 miRNAs clearly separate AD and MCI-AD samples from control samples both in pilot and verification studies with regards to specificity, sensitivity and area under the curve (AUC) values given for each miRNA in Table 6. Furthermore, Figure 5 shows ROC curves of two selected best exampled miRNAs: hsa-miR-483-5p, the novel AD biomarker candidate, for which the fold increase was extremely high when comparing MCI-AD and AD patients to non-demented controls (8-13 fold change), and hsa-miR-502-3p, the previously reported candidate which represents miRNA of lower fold increase in AD and MCI-AD patients compared to controls (approx. 2 fold change). Table 6 and Figure 5 indicate that these two miRNAs separated AD patients from controls, as well as MCI-AD patients (early AD) from controls, with AUC over 0.9, specificity and sensitivity over 0.80, repeatedly in both pilot and verification studies. Thus, based on consistently high ROC curve parameters, the miRNAs hsa-miR-483-5p and hsa-miR-502-3p represent the most promising AD biomarkers.

**Figure 6:**
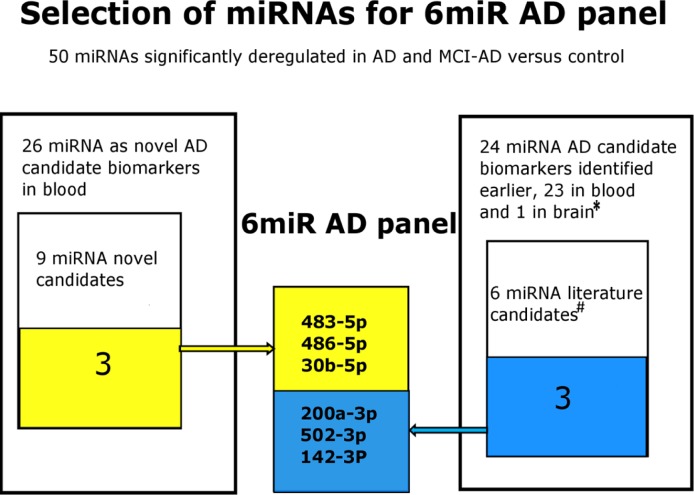
The selection process scheme of the 6miR panel differing early AD from control plasma The 6miR panel was selected in this study based on the qRT-PCR pilot screening of Exiqon plasma-focused panel of 179 miRNAs followed by comparison of the results with the all previously reported miRNAs in AD blood (31 articles)* and by qRT-PCR verification of 9 novel and 6 previously reported miRNAs as candidate AD biomarkers. The verification experiment indicated 6 miRNAs as the best candidates for further verification in larger cohorts as possible early AD biomarkers. References: * [20–46, 57, 84–87], # [21, 22, 28, 30, 57, 87].

Finally, we compared all analyzed parameters of pilot and validation studies for the selection of the most promising biomarker candidates for diagnosis of early AD in plasma of MCI patients. Table 7 shows the selected 6 best miRNAs including 3 novel biomarker candidates based on the highest fold changes, consistent statistical significance of changes, and the highest specificity and sensitivity values of separation of non-demented controls from early AD subjects. This makes the optimum set of 6 miRNAs for diagnosis of early AD (6miR). The scheme of the selection process of this 6miR panel in this study is shown in Figure 6.

**Table 7:** miRNAs selected after preliminary and verification experiments as most promising plasma biomarker candidates for the detection of early AD in MCI patients

miRNAs (hsa-miR)	MCI-AD vs CTR									
	D1	D2	F1	F2	Auc1	Auc2	SP1	SP2	SN1	SN2
**483-5p**	↑**	↑***	**5.58**	**14.72**	**0.93**	**0.95**	**1**	**1**	**0.83**	**0.87**
486-5p	↑	↑***	1.72	4.39	0.90	0.87	1	0.89	0.86	0.87
30b-5p	↓**	↓	1.45	1.45	0.95	0.82	0.83	0.89	1	0.87
**200a-3p**	↑*	↑*	**4.00**	**3.07**	**1**	**0.83**	**1**	**0.89**	**1**	**0.75**
502-3p	↑	↑**	1.65	2.25	0.90	0.86	0.83	0.89	0.86	0.87
142-3p	↓**	↓	1.8	1.32	1	0.80	1	0.78	1	0.87

D-Directionality; F- Fold change; ROC curve analysis: Auc- Area under the ROC curve, SP- Specificity, SN-Sensitivity. D1, F1, Auc1, SP1 and SN1 in MCI-AD1 *vs* CTR1 and D2, F2, Auc2, SP2 and SN2 in MCI-AD2 *vs* CTR2. miRNAs previously reported as AD biomarker candidates in the blood and verified here are shaded in grey; remaining are miRNAs identified here as novel AD biomarker candidates. Stars denote statistical significance (one-way ANOVA with post-hoc Tukey HSD p < 0.05). miRNAs marked in bold are significant in both the preliminary and verification experiments and the remaining miRNAs are markedly significant in at least one stage (pilot or verification).

### Predicted target transcripts related to Alzheimer’s pathomechanism

We investigated the potential functions and involvement in AD pathomechanisms of the 15 miRNAs selected in the pilot screening. Table 8 presents results of *in silico* search performed with Targetscan 7.1 software for identifying target mRNAs among the mRNAs encoding key proteins of the amyloid cascade and tau protein. Importantly, this analysis showed that such crucial AD proteins as APP, BACE 1 and tau (MAPT) can be regulated by more than one miRNA of the investigated 15 miRNAs. Of note, as much as 4 of the 15 miRNAs have binding sites in APP mRNA. Moreover, hsa-miR-483-5p, which we found as one of the most deregulated miRNAs and the best biomarker candidate for early AD detection, has a binding site in tau mRNA, together with 4 other miRNAs of the 15 biomarker candidates. Since miRNAs are known to function in complementary regulatory networks, the multiple binding sites in mRNAs closely related to AD pathology for several miRNAs out of the 15 differentiating AD from controls suggests that these miRNAs may be relevant in AD pathogenesis.

**Table 8:** Predicted binding sites in AD-related transcripts for the differential 15 miRNAs in AD plasma

Target gene	miRNAs	miRNA and their seed region matching site in the 3′UTR region of gene
**APP**	hsa-miR-1260a hsa-miR-320a/b/c	hsa-miR-1260a : 80-86 hsa-miR-320a/b/c : 2355-2361 2743-2750
**BACE 1**	hsa-miR-200a-3p hsa-miR-1260a hsa-miR-320a/b/c	hsa-miR-200a-3p : 1551-1558 hsa-miR-1260a : 1559-1565 hsa-miR-320a/b/c : 3216-3222
**BACE 2**	hsa-miR-483-5p	hsa-miR-483-5p : 103-109 3798-3804
**MAPT (Tau)**	hsa-miR-151a-3p hsa-miR-483-5p hsa-miR-320a/b/c	hsa-miR-151a-3p : 101-107 hsa-miR-483-5p : 1143-1149 hsa-miR-320a/b/c : 2795-2801 3885-3891
**PSEN2**	hsa-miR-30b-5p	hsa-miR-30b-5p : 121-127

Search was performed with Targetscan 7.1 software. APP- Amyloid Beta Precursor Protein; BACE 1- Beta-Site Amyloid Precursor Protein Cleaving Enzyme 1; BACE 2- Beta-Site Amyloid Precursor Protein Cleaving Enzyme 2; MAPT- Microtubule-Associated Protein Tau (Tau Protein); PSEN2- Presenilin 2.

**Figure 7:**
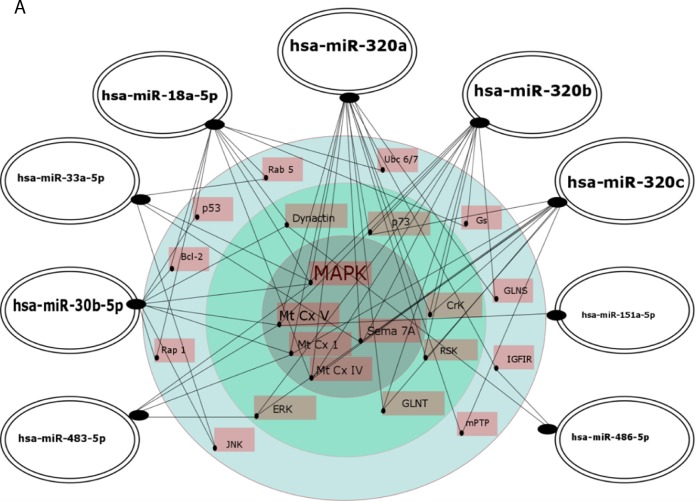

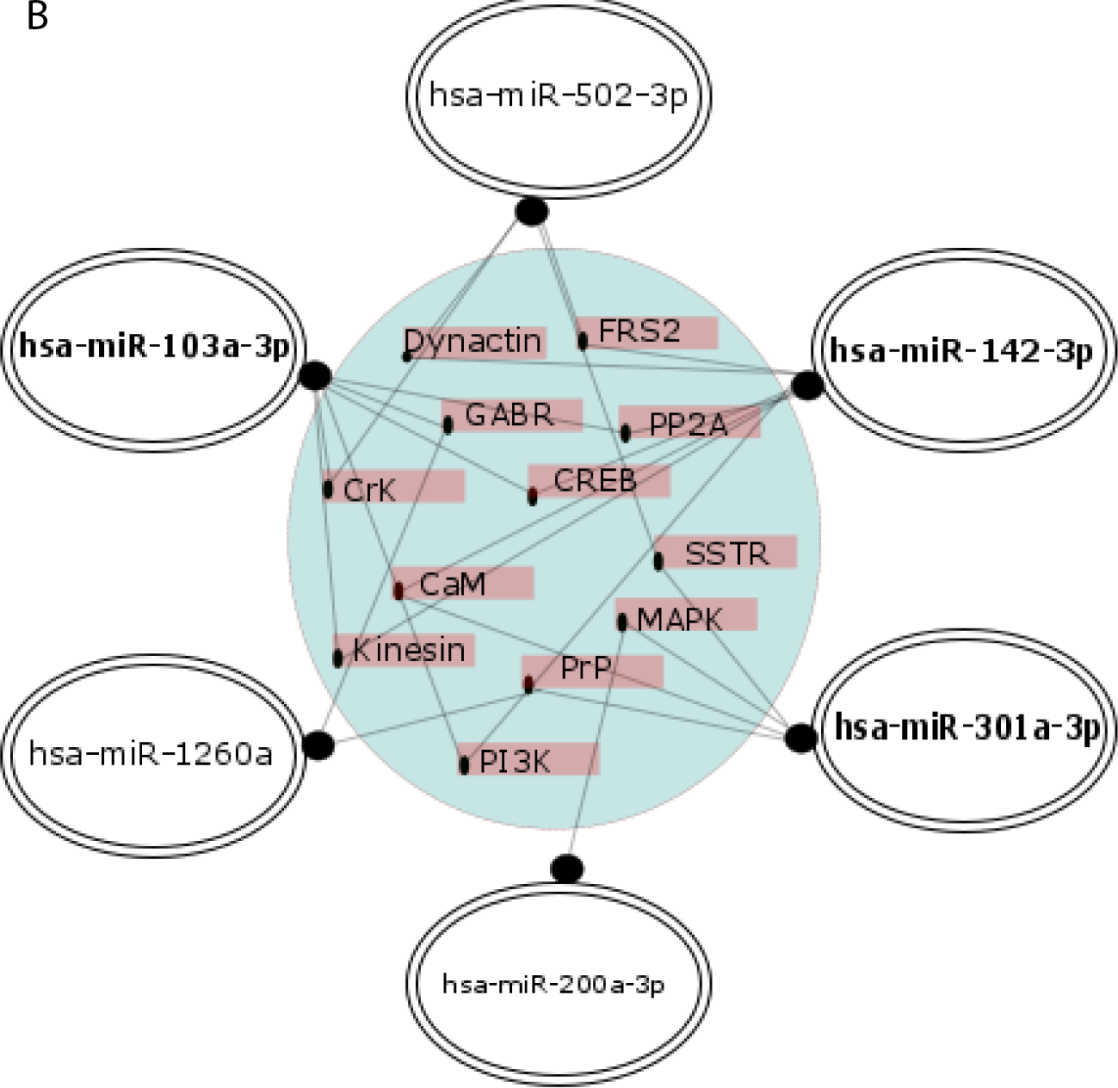
Regulatory network of 9 novel (**A**.) and 6 previously reported (**B**.) AD biomarker candidate miRNAs and their putative cellular effectors. Putative targets were identified by searching the MirTarBase (version 6) database which contains experimentally validated miRNA target genes followed by KEGG’s database search for pathways contributing to neurodegenerative diseases and pathways of the nervous system. For construction of miRNA regulatory network only target genes/proteins that had 2 or more hits were considered. In A, the inner circle contains targets common for at least 4 up to 6 miRNAs, the middle layer contains targets common for 3 miRNAs, and the outer layer contains targets for 2 miRNAs. In B, targets shown are for 2 miRNAs. Highlighted are the miRNAs with higher number of hits to putative protein targets.

A group of potential downstream target genes/proteins known to contribute to AD pathogenesis was also identified in an independent approach based on searching the MiRTarBase, comprising experimentally validated miRNA target genes, followed by searching the KEGG database for signaling pathways in neurodegenerative diseases and in the nervous system regulated by the analyzed 15 miRNAs, including 9 novel AD biomarker candidates (Figure 7, Supplementary Table 2). The target proteins were grouped from the ones regulated by 6 miRNAs down to the ones regulated by 2 miRNAs of the 15 miRNAs. Common targets for the 9 novel miRNAs were found for maximum of 6 miRNAs, while common targets for previously reported differential miRNAs were found for a maximum of two miRNAs. As shown in Figure 7, the analysis indicated a network of targets centered around the mitochondria respiratory chain that were implicated in oxidative stress in AD pathology by a vast number of independent reports, as recently reviewed [58, 59]. MAPK are also known to be in center stage of aberrant cellular signaling in AD pathology [59]. Potential downstream effectors regulated by 3 or 2 of the analyzed miRNAs include other proteins known to contribute to AD pathogenesis, such as the insulin growth factor receptor (IGFR I) [60, 61], apoptosis-related proteins such as p53, Bcl-2 [62–64], and proteins involved in endocytosis and intracellular signaling (Rab5, ERK) [65, 66]. The search also identified multiple target genes regulated by only one miRNA of the panel known to be related to AD pathology, such as BACE (Supplementary Table 2).

## DISCUSSION

The development of AD diagnostics relies on two major concepts: progressing from assessment of only neuropsychological symptoms to combined assays involving molecular AD signatures, and minimizing invasiveness and increasing availability of the diagnostic methods. In particular, development of reliable, non-invasive methods for diagnosing early AD in MCI patients is considered to be crucial for increased efficiency of available therapeutic treatments, as well as for proper recruitment of patients for clinical trials of novel drugs, and for monitoring disease progression and response to treatment. Blood-based, stable, molecular biomarkers such as miRNAs seem to respond well to criteria of future AD biomarkers, but so far miRNA signatures in early AD patients are uncertain.

Here we report the discovery of a 6 miRNA profile (6miR) in plasma which separated non-demented control subjects from MCI patients diagnosed with probable early AD supported by CSF biomarker assays. The results suggest that the 6miR in the blood are promising candidates that should be verified in future studies in larger cohorts as a possible replacement of invasive CSF markers as identifiers of early AD (patent pending, PCT/IB2016/052440). The selected 6 best candidate biomarkers for early AD are among the top ones in the differential panel of 15 miRNAs obtained in the pilot screening which were functionally mapped to proteins involved in AD pathology by the two independent *in silico* searches in databases containing predictive (TargetScan) and validated (MirTarBase) miRNA targets.

### Notes on study design

We applied qRT-PCR as the method most sensitive for quantitative assessment of levels of well-defined miRNAs, and most suitable for translation to clinical practice, as reviewed [67]. The source of miRNAs is another important aspect. So far different blood sources were investigated for detection of circulating miRNAs in AD patients: plasma [21, 31, 34, 35, 38, 46, 87], serum [20, 24, 25, 27, 32, 33, 36, 40–43], blood mononuclear cells [30], whole blood [22, 84–86] and plasma exosomes [29]. All miRNA sources have some advantages and disadvantages. We have chosen plasma, because plasma is readily available in clinical settings, can be easily assessed for elimination of hemolytic samples, and standardized plasma collection with EDTA as an anticoagulant minimizes the effects on qRT-PCR amplification. Moreover, coagulation for gaining serum leads to increased expression and release of miRNAs from platelets. Applying qRT-PCR analysis of plasma samples, this study provided the first broader, systematic, independent confirmation of multiple miRNAs reported earlier, using NGS or qRT-PCR as AD candidate biomarkers in blood cells, blood plasma, whole blood and also in AD brain. This data suggests that qRT-PCR of plasma could be further developed as a diagnostic test for AD.

Some MCI patients may suffer from early AD and may later develop AD dementia, while others may not. Therefore the lack of assessment of AD molecular correlates such as classical CSF biomarkers leads to doubts as to the homogeneity and identity of the MCI groups, and makes interpreting the results difficult. In fact, there is a discrepancy in existing data of blood-based miRNAs in MCI patients because MCI was either considered as a dementia type/stage different than AD [22, 23, 39, 43] or MCI was assumed to represent early AD, and was included in the analysis together with probable AD as one disease group [20, 21]. Some other authors attempted to stratify the MCI group according to dementia psychological assays and PIB-PET brain imaging, but the classification omitted CSF markers and thus cannot be referred to such biochemical measures [41, 68–70]. To avoid such problems, our study involved only those patients diagnosed with MCI, in which analysis of AD biomarkers in CSF indicated likelihood of early AD. To increase group identity and homogeneity, and to provide some selective pressure for AD specificity, we included in our analysis only patients with the diagnosis of MCI due to AD. We have resigned on the involvement of MCI patients with low CSF markers, because the later development of AD in such MCI patients is not excluded and the data would be inconclusive. Instead, we compared patients with early AD (MCI-AD) to patients with moderate AD (AD), according to CSF biomarker levels and classical dementia ratings, and to two age-matched non-demented control groups. In our study we relied on the lack of any clinical features of MCI or dementia in the control cohort. While it would be optimal to exclude preclinical AD in this group, at present there are no consensus tests to exclude probability of AD development over time in asymptomatic subjects [8].

When compared to healthy individuals, classical CSF biomarkers have a high predictive accuracy. However, CSF biomarkers have been proved less accurate for separating AD from other dementias. To avoid difficulties in interpreting data referring to CSF biomarker levels, in this study we resigned on recruiting patients with other dementias.

Although our study enabled direct comparison of miRNAs in plasma to levels of AD CSF markers, we found no correlation of the levels of any of the 15 miRNAs analyzed both in the pilot and verification screenings with any of the conventional AD CSF markers (not shown). This result is in agreement with 3 other reports in AD in which no correlation of blood miRNAs with CSF miRNA AD markers were found [27, 31, 38]. Instead, profiles of miRNAs in blood plasma were found to correlate with disease status [27]. Lack of correlation of our plasma miRNA biomarker candidates with CSF AD markers seems understandable in light of a body of literature. It is known that miRNAs in plasma can come from other tissues including brain cells and also directly from blood cells [71–73]. Moreover, the systemic nature of AD pathology involving molecular alterations in blood cells was demonstrated in several studies by others and ourselves [58, 64, 74–76]. This data altogether suggests that blood-based miRNA biomarkers should be seen as a molecular signature of complex AD pathology of a systemic nature, rather than only the reflection of AD pathological hallmarks in the brain or CSF.

### Confirmation of previously reported differential miRNAs in AD blood

One of major needs in research concerning miRNAs as biomarkers in AD blood is verification of already identified candidate biomarkers in different cohorts of patients. Importantly, our study of the Polish subjects provided independent, standardized confirmation for 23 miRNAs previously identified as AD biomarker candidates in blood, and verified two out of them as the best plasma biomarkers for early AD: hsa-miR-200a-3p and hsa-miR-502-3p. In spite of variations in the cohorts of independent studies, such as cognitive status, geographical area and lifestyle, or genetic factors like apolipoprotein E, we observed 23 differential miRNAs in early (and later, moderate) AD, that overlapped in the present study with previous studies of AD miRNAs in blood (Figure 2). Out of the 23 miRNAs, 10 miRNAs were common for early AD (MCI due to AD) and developed AD. It should be highlighted that out of the 10 miRNAs, 4 miRNAs were earlier reported in two different studies: hsa-let-7f-5p [22, 30], hsa-let-7d-5p [21, 24], hsa-let-7g-5p [21, 28] and hsa-miR-142-3p [21,87]. Other miRNAs were reported in single studies: hsa-miR-301a-3p [21], hsa-miR-103a-3p and hsa-let-7d-3p [22], hsa-miR-200a-3p [30], hsa-miR-15b-3p [41], hsa-miR-502-3p [28]. Moreover, hsa-miR-22-5p [27], hsa-miR-186-5p and hsa-miR-30c-5p [28] were found significantly deregulated only in patients with later AD, and the remaining 10 miRNAs were found in early AD (MCI-AD) patients: hsa-miR-342-3p [24, 41], hsa-miR-146a-5p [34, 43], hsa-miR-106a-5p [41, 84], hsa-miR-339-3p and hsa-miR-339-3p [37], hsa-miR-18b-5p [41], hsa-miR-338-3p [29], hsa-miR-660-5p [28], hsa-miR-150-5p [29], hsa-miR-27b-3p [39]. Thus, our results strongly support the potential of miRNAs as plasma-based AD biomarkers in AD, assuming strict standardization of methodology.

On the other hand, some of the miRNAs reported earlier as potential AD biomarkers were not confirmed in our study. This partial lack of reproducibility may reflect either the biological heterogeneity of the human populations and of AD subphenotypes and endophenotypes, or the variability of methodological approaches and diagnostics measures for recruiting patients in various studies. As discussed recently by other authors, even in the same study, based on the same methodological approach to miRNAs analysis, the difference in mean age of the two compared AD patient cohorts was probably a reason for the lack of consistency in the expression levels of the two analyzed potential AD biomarkers, miRNAs miR-34a-5p and miR-545-3p [87]. More studies are needed to evaluate the possible application limits of miRNAs as AD biomarkers, such as differences in methodology, as well as factors beyond methodology.

### The best candidates for future testing as potential plasma biomarkers in early AD: implication in AD pathogenesis

Our study indicated 15 miRNAs which could be further evaluated as potential plasma biomarkers for early AD, including 9 novel candidates. Out of the 15 miRNAs, the 6miR panel was selected based on the verification screening as the best plasma biomarker candidates for early AD, according to best characteristics, including best specificity and sensitivity (Figure 6, Table 7). The 6miR consists of 3 novel candidate biomarkers and 3 previously reported differential miRNAs in AD.

3 miRNAs of the 6miR panel were reported earlier as AD biomarker candidates: hsa-miR-200a-3p, hsa-miR-502-3p and has-miR-142-3p. Increased levels of hsa-miR-200a were earlier reported in AD blood mononuclear cells [30]. Our report shows 3 to 4 fold increased plasma levels of hsa-miR-200a-3p in AD, suggesting that this miRNA can be secreted by blood mononuclear cells. Our search for predicted transcripts regulated by hsa-miR-200a-3p identified a binding sequence in the 3′UTR of BACE1 mRNA, one of the key enzymes involved in the amyloidogenic proteolysis of APP. hsa-miR-200a-3p may be thus involved in the regulation of BACE1 in the brain or in blood cells which are known to express BACE1, possibly as a compensatory mechanism in AD. hsa-miR-200a-3p may also be linked to other regulatory pathways contributing to AD. Among predicted target genes/proteins or protein complexes for hsa-miR-200a-3p is the CxV protein of the mitochondrial oxidative chain, and also p53 and p38 signaling proteins. Interestingly, a feedback loop among p38/p53 and hsa-miR-200a-3p was described, in which the p53-mediated expression of hsa-miR-200a-3p promoted cell death [77]. Indeed, in an independent report hsa-miR-200a-3p was found to be involved in cell cycle inhibition and apoptotic induction [78]. In agreement with this data, several studies by others and ourselves demonstrated a deregulated cell cycle and apoptosis in blood mononuclear cells in AD, associated with p53 signaling [58, 64, 74–78]. Thus hsa-miR-200a-3p seems to be one of the regulatory factors involved in the altered cell cycle regulation in blood mononuclear cells in AD, supporting the cell cycle hypothesis as a component of AD pathogenesis.

In turn, hsa-miR-502-3p was found consistently upregulated in early AD, and with high specificity and sensitivity separating early AD plasma samples from controls. In contrary, decreased levels of hsa-miR-502-3p were reported in whole blood [28]. The opposite direction of the miRNA deregulation in our report is the only difference in the direction of changes between our study and the earlier reports. This discrepancy in the hsa-miR-502-3p direction of change in AD plasma *versus* whole blood can be attributed to several methodological differences between the two studies. hsa-miR-502-3p is of interest as an early AD biomarker because differential expression of blood plasma hsa-miR-502-3p was also reported in obstructive sleep apnea, the condition known to promote AD [79, 80]. Furthermore, a confirmed target for this miRNA is linked to cell cycle regulation [81], in agreement with the cell cycle hypothesis of AD pathogenesis supported by several studies including our recent reports, as mentioned above [58, 64, 74–78]. Other putative target proteins identified by our database search for hsa-miR-502-3p are dynactin (dynein activator complex) and superoxide dismutase 2 (SOD2). Both proteins were implicated in AD pathogenesis by multiple studies: SOD2 as one of the key mitochondrial anti-oxidative stress proteins clearing reactive oxygen species (reviewed in [82]), and dynactin as a known complex regulator of dynein binding to cell vesicles to be transported along microtubules, which contributes to tau-induced toxicity [83].

Out of 9 miRNAs for the first time indicated as potential biomarkers for early AD in this report, hsa-miR-486-5p and hsa-miR-483-5p are particularly interesting based on the very good biomarker characteristics, and exceptionally high fold changes when compared to so far reported values for differential miRNAs in AD (Table 7). In our study miR-483-5p showed the highest fold change upregulation in early AD in MCI patients (about 13 fold increase in the verification experiment), and also in later, moderate AD patients. Furthermore, our searches of TargetScan, MirTarBase and KEGG databases identified hsa-miR-483-5p as a potential regulator of several key proteins/signaling pathways involved in AD pathology. TargetScan indicated hsa-miR-483-5p binding site in tau protein, together with binding sites for 4 other of the 15 analyzed miRNAs, and in the BACE protein. BACE as its putative target was also indicated in the MirTarBase/KEGG databases. According to the network hypothesis of miRNAs regulatory functions, the MirTarBase/KEGG search pointed to 4 common target proteins for 3-6 miRNAs of the 15 miRNA panel, including hsa-miR-483-5p: MAPK, proteins of the mitochondrial oxidative chain complexes Mt CxI and Mt CxV, and ERK (Table 8, Supplementary Table 1, Figure 7). In turn, hsa-miR-486-5p together with hsa-miR-30b-5p and hsa-miR-18a-5p proved to be among the putative regulators of dynactin, similarly as described for hsa-miR-502-3p above. hsa-miR-486-5p was also found to possibly regulate IGFRI, together with hsa-miR-320a.

In overall, the miRNAs of the selected 6miR as potential diagnostic panel for early AD are among the top ones of 15 investigated miRNAs which were functionally mapped to proteins involved in AD pathology by our two different bioinformatics searches in the databases containing predictive (TargetScan) and experimentally validated (MiRTarBase) miRNA targets. The 15 biomarker candidate miRNAs showed common (from 6 to 2 miRNAs) functional effectors among proteins directly related to AD pathology, such as APP, BACE, MAPT, PSEN2, as well as other proteins known to contribute to AD, such as proteins of the mitochondrial oxidative chain, cell cycle and cell fate kinases MAPK and ERK, cell cycle and apoptosis regulatory proteins p53 and Bcl-2, and IGFRI. This data supports the role of miRNAs in network regulation and contribution to AD of several signaling pathways, according to the cell cycle hypothesis, mitochondrial hypothesis and the AD as type 3 diabetes hypothesis, postulated by mounting results of others and ourselves [58, 64, 74–78].

In summary, the analysis provided consistent data in support of the 6miR panel differentiating early AD from control blood plasma, and proposed 6 early AD biomarker candidates for future testing in larger cohorts. We put special emphasis on standardized diagnostics, including CSF biomarker assessment and detailed blood handling procedures, according to the internationally accepted protocols, and we applied commercially available Exiqon qRT-PCR assays. The standardization of methodology opens a possibility for comparability and multicenter experimental verification of the identified miRNAs as AD biomarker candidates. The lack of such standardization is probably, to a high degree, responsible for the lack of broader consistency between miRNA biomarker candidates identified in AD patients in different studies. Verification of the candidate biomarker miRNAs in other larger cohorts of AD patients, compared to other neurodegenerative and neurological diseases, as well as a follow up study of the MCI-AD patient cohorts investigated in this report, would be necessary as a classical next step in the development of future potential diagnostics of early AD.

## CONCLUSIONS

Our findings support the concept of miRNAs as potential plasma-based AD biomarkers in AD. However, our results point to the necessity of determining which miRNAs identified so far as potential AD biomarkers are sensitive to methodological variability, and which to factors other than methodological, including AD subphenotypes. Nevertheless, it seems that circulating miRNAs such as indicated and verified in our study indeed form a molecular signature already present in the early AD stage, and thus can replace invasive CSF markers. In the future, miRNA profiles hold promise for differentiating AD stages, allowing the monitoring of disease progression. The next step required for the development of blood-based diagnostics of AD will also be the harmonization of novel biomarker candidates, including circulating miRNAs, as well as other biomarker molecules in blood cells and serum, with all the existing diagnostic tools, in order to determine and adjust the cut-off range of the multivariate diagnostic panel. Moreover, an analysis of the putative functional targets of miRNAs and their future experimental validation can provide input in deciphering AD pathogenesis.

## MATERIALS AND METHODS

### Study subjects

All subjects in the study were Caucasians from Poland. Blood samples were obtained from patients enrolled in the Alzheimer’s Ward of the Central Clinical Hospital of the Ministry of Interior (MSW) in Warsaw. Experimental protocols used for obtaining and analyzing blood plasma samples were approved by the Ethics Committee for Studies on Human Subjects at the Central Clinical Hospital of the Ministry of Interior in Warsaw, Poland (agreement no 89/2014), and are in compliance with National and European Union legislation, and the Code of Ethical Principles for Medical Research Involving Human Subjects of the World Medical Association. Peripheral blood samples were collected from all subjects after written informed consent was obtained from the patients or their legal representatives. All clinical diagnoses were performed according to the criteria of the Diagnostic and Statistical Manual of Mental Disorders, 4th edition (DSM-IV), the NINCDS-ADRDA (Criteria of National Institute of Neurological and Communicative Disorders and Stroke and the Alzheimer’s Disease and Related Disorders Association) and according to the recommendations from the National Institute of Ageing - Alzheimer’s Association workgroups on diagnostic guidelines for Alzheimer’s disease [6, 7]. The patients were diagnosed by neurologists, neuropsychologists, and other qualified staff members in the clinic. Diagnosis was based on an interview, the MMSE test (an assay of global cognitive impairment, scale 0-30), the Clinical Dementia Rating (CDR) test (an assay able to distinguish very mild dementia; dementia scale: 0 - none, 0.5 - very mild, 1 - mild, 2 - moderate, 3 - severe), and radiological tests (MRI) of the hippocampus. The AD diagnosis of patients recruited for our study was additionally supported by the results of CSF assays based on the levels of standard markers: Aβ peptide, t-tau (“total” tau protein) and p-tau (phosphorylated form of tau protein). The CSF assays were carried out in details as recommended by the international JPND BIOMARKAPD consortium. AD biomarker concentrations were examined by using a sandwich enzyme-linked immunosorbent assay kit (ELISA) (Innogenetics, Gent, Belgium) in the hospital laboratory. For detection of p-tau, the INNOTEST^®^ PHOSPHO-TAU(181P) was used. Cut off values of assayed CSF markers for AD diagnosis were Aβ42 < 609.54 pg/ml, t-tau > 277.02 pg/ml and p-tau > 55.08 pg/ml, as described previously in [88]. Patients’ characteristics are presented in Table 1. The group of patients denoted as AD (AD1 and AD2) comprised 20 patients with clear evidence of a developed AD pathological process according to dementia indicators (CDR values > 1), MMSE results > 11 < 26, and according to levels of CSF markers (Table 1). The MCI group with a likelihood that the MCI was due to AD was denoted MCI-AD (MCI-AD1 and MCI-AD2) and comprised 15 patients in the early AD stage, as confirmed by levels of AD CSF biomarkers, but with lower dementia indicators (CDR rating = 0.5), and MMSE results > 21 < 29. According to the updated research criteria for the diagnosis of MCI due to AD [7], patients with positive CSF tests for both Aβ peptide and CSF tau/p-tau show the highest possible likelihood of MCI due to AD. In our study 8 MCI patients fulfilled these criteria: 4 in MCI-AD1 group and 4 in MCI-AD2 group (Table 1). Positive markers of neuronal injury alone (CSF levels of tau/p-tau) are still sufficient for diagnosing the likelihood of MCI due to AD [7]. In the present study 7 MCI patients were positive for t-tau and p-tau: 3 patients in MCI-AD1 group and 4 patients in MCI-AD2 group (Table 1). Moreover, MRI of the hippocampus additionally showed AD-type changes in all the MCI-AD patients enrolled in the study.

The two control groups comprised age-matched subjects, 8 females and 7 males aged 60-70 without dementia; one group consisted of 6 attendees of the University of the Third Age, selected as a group of people at low risk of dementia due to intellectual activity, and not taking any medication. The second control group consisted of 9 non-dementia patients enrolled in the Independent Public Central Clinical Hospital at Banacha street in Warsaw, but without any memory problems and with no family history of AD. The control groups included individuals without dementia; exclusion criteria comprised of neurodegenerative diseases (Alzheimer’s, Parkinson’s, Huntington, FTD, Tauopathies, etc.), memory impairment (MCI, subjective cognitive impairment, SCI), a history of familial AD, diabetes type 1 and 2, cancer and neurological disorders (schizophrenia, autism, depression, etc.). In overall, the population in the study was well-balanced according to sex and age, with no significant differences between the cohorts.

### Blood collection and sample handling

Blood samples from all study subjects were withdrawn by venipuncture into BD Vacutainer tubes with EDTA and a gel separating plasma from blood cells to avoid hemolysis. The samples were centrifuged immediately. Aliquots of plasma were stored in -80°C. The procedure was carried out in detailed adherence to SOPs (Standard Operating Procedures) established under the international JPND BIOMARKAPD project, and in agreement with Exiqon’s recommendations.

### RNA isolation

RNA isolation from collected blood plasma samples was performed using the miRCURY^TM^ RNA Isolation Kit - Biofluids (Exiqon) according to the manufacturer’s recommendation. Briefly, RNA isolation controls (UniSp2, UniSp4 and UniSp5) were added to the purification step to detect any differences in extraction efficiency. Isolated RNA was stored at −80°C until use and transported in dry ice.

### Quantitative real time-PCR (qRT-PCR)

Synthesis of cDNA and qRT-PCR was performed according to “miRCURY™ microRNA QC PCR Panel - Instruction manual v1.1”, as instructed by Exiqon. All miRNAs were reverse transcribed into cDNA in a single reaction step. The cDNA synthesis control (UniSp6) was added in the reverse transcription reaction giving the opportunity to evaluate the RT reaction. cDNA and Exilent SYBR Green mastermix were transferred to the qPCR panel preloaded with primers (Exiqon), using a pipetting robot. In the pilot experiment, a qRT-PCR-based commercially available Exiqon panel for assessment of blood plasma levels of 179 miRNAs was used. The 179 miRNAs on the panel were selected based on the data collected in the Exiqon database. In the verification experiment, a custom-made miRNA panel was employed. Amplification was performed in a Roche Lightcycler 480. Raw Cp values and melting points, as detected by the cycler software, were exported. Reactions with several melting points, or with melting points that are not within assay specifications, were flagged and removed from the data set. Reactions with an amplification efficiency below 1.6 were removed as well. Reactions giving Cp values within 5 Cp values of the negative controls, were removed from the dataset. Moreover, all the data was normalized to correct for potential overall differences between samples. A “no template” sample in the RT step was included as a negative control.

An additional step in the real-time PCR analysis was performed to evaluate the specificity of the amplification products by generating a melting curve for each reaction. A major source of variation in plasma samples is contamination with potential cell-derived miRNA, especially from hemolysis [49]. Only samples that showed no signs of hemolysis were analyzed (according to the ratios of hsa-miR-451 which is expressed in RBC to hsa-miR-23a which is stable serum miRNA).

### Bioinformatics and statistical analysis

Exiqon qRT-PCR panel normalization in the pilot study was performed based on the average of the assays detected in all samples, as this was shown to be the best normalization for qPCR studies involving numerous assays [50]. The normalization included 153 assays out of the 179 miRNAs analyzed in the pilot study. 153 miRNAs were identified in all samples, with an average of 170 miRNAs detectable per sample. As measured by the NormFinder software [51], the stabilities of the average of 153 miRNAs in the pilot study were higher than those of any single normalizer miRNA in the data sets. For normalization of the data in the verification experiment, we selected 5 normalizer miRNAs which were most stable in the pilot experiment: hsa-miR-185-5p, hsa-miR-128-3p, hsa-miR-130b-3p, hsa-miR-15a-5p and hsa-miR-425-3p, and used the average of the 5 normalizer assays. The formula used to calculate the normalized Cq values: normalized Cq = average Cq (n) - assay Cq (sample). A higher value thus indicates that the miRNA is more abundant in that particular sample.

Statistical significance of the comparison of changes in miRNAs levels between subject groups are given based on the one-way ANOVA with post-hoc Tukey test HSD (honestly significant difference) unless specifically mentioned. Normal distributions of data for miRNAs levels in all groups in the pilot and verification studies were checked using the Shapiro-Wilk test. The post-hoc *p*-values shown in this report were for multiple comparisons. They are, according to Prism and InStat standards, as follows: not significant (ns) *p* > 0.05, * *p* < 0.05, ** *p* < 0.01, *** *p* < 0.001, **** *p* < 0.0001.

The values of receiver operating characteristic (ROC) curves including the area under the ROC curve (AUC) of individual miRNAs were plotted and calculated using the pROC package in R environment as described previously [52]. The specificity and sensitivity values were calculated for the balanced accuracy point (optimal operating threshold) on ROC curves.

Target genes for miRNAs were obtained from two different database analysis. The TargetScan database (release 7.1) [53] was used to explore possible/predicted miRNA-mRNA interactions to identify target mRNAs among the mRNAs encoding tau protein and key proteins of the amyloid cascade. In addition, the MirTarbase (version6.0) database which contains experimentally validated miRNA target genes [54] was used to explore miRNA gene targets which were subsequently mapped to KEGG pathways [55]. Pathways which contribute to neurodegenerative diseases (KEGG IDs: 05010-Alzheimer’s disease, 05012-Parkinson’s disease, 05014-Amyotrophic lateral sclerosis, 05016-Huntington’s disease and 05020-Prion disease pathways) and pathways in the nervous system (KEGG IDs: 04080-Neuroactive ligand-receptor interaction, 04360-Axon guidance, 04724-Glutamatergic synapse, 04727-GABAergic synapse, 04725-Cholinergic synapse, 04728-Dopaminergic synapse, 04726-Serotonergic synapse, 04720-Long-term potentiation, 04730-Long-term depression, 04723-Retrograde endocannabinoid signaling, 04721-Synaptic vesicle cycle, 04722-Neurotrophin signaling pathway) were analyzed for miRNA targets. Mapping of miRNAs to the gene targets in pathways was performed with the R/Bioconducor pathview package [56].

### Literature search criteria

Search terms of “MicroRNA” or “miRNA” AND “Blood” AND “Alzheimer disease” in Web of Science (Collection of Databases: Web of Science^TM^ Core Collection, Current Contents Connect, Data Citation Index^SM^, BIOSIS Citation Index^SM^, Derwent Innovations Index^SM^, KCI-Korean Journal Database and MEDLINE) shortlisted 193 articles as of 1^st^ September 2016. Based on exclusion criteria (review, conference articles and animal model studies), and based on inclusion criteria (only microRNA studies in whole blood, blood cells, plasma and serum) 31 articles were found to be eligible to perform comparative analysis with our stage 1 results [20–46, 84–87].

In our study, primers for mature sequence miRNAs (3p/5p arm maturations) were used. When primers used in the previous studies were not for mature sequences, primers used for parent stem loop were considered and that particular miRNA was included as reproduced miRNA. This is the case for the following miRNAs in our study: hsa-miR-200a-3p, hsa-let-7f-5p, hsa-miR-146a-5p, hsa-miR-339-5p, hsa-miR-339-3p, hsa-miR-27b-3p. The previously reported miRNAs were, respectively: hsa-miR-200a [30], hsa-let-7f [30], hsa-miR-146a [34, 43], hsa-miR-339 [37], hsa-miR-27b [39].

## SUPPLEMENTARY FIGURE AND TABLES





## References

[1] Alzheimer Disease International, Dementia statistics http://www.alz.co.uk/research/statistics.

[2] Hurd MD, Martorell P, Delavande A, Mullen KJ, Langa KM Monetary Costs of Dementia in the United States. N Engl J Med.

[3] Jack CR, Knopman DS, Jagust WJ, Peterson RC, Weiner MW, Aisen PS, Shaw LM, Vemuri P, Wiste HJ, Weigand SD, Lesnick TG, Pankratz VS, Donohue MC, Trojanowski JQ Tracking pathophysiological processes in Alzheimer’s disease: An updated hypothetical model of dynamic biomarkers. Lancet Neurol.

[4] Gelosa G, Brooks DJ The prognostic value of amyloid imaging. Eur J Nucl Med Mol Imaging.

[5] Devanand DP, Liu X, Tabert MH, Pradhaban G, Cuasay K, Bell K, de Leon MJ, Doty RL, Stern Y, Pelton GH Combining early markers strongly predicts conversion from mild cognitive impairment to Alzheimer’s disease. Biol Psychiatry.

[6] McKhann GM, Knopman DS, Chertkow H, Hyman BT, Jack CR, Kawas CH, Klunk WE, Koroshetz WJ, Manly JJ, Mayeux R, Mohs RC, Morris JC, Rossor MN The diagnosis of dementia due to Alzheimer’s disease: recommendations from the National Institute on Aging-Alzheimer’s Association workgroups on diagnostic guidelines for Alzheimer’s disease. Alzheimers Dement.

[7] Albert MS, DeKosky ST, Dickson D, Dubois B, Feldman HH, Fox NC, Gamst A, Holtzman DM, Jagust WJ, Petersen RC, Snyder PJ, Carrillo MC, Thies B, Phelps CH The diagnosis of mild cognitive impairment due to Alzheimer’s disease: recommendations from the National Institute on Aging-Alzheimer’s Association workgroups on diagnostic guidelines for Alzheimer’s disease. Alzheimers Dement.

[8] Dubois B, Hampel H, Feldman HH, Scheltens P, Aisen P, Andrieu S, Bakardjian H, Benali H, Bertram L, Blennow K, Broich K Preclinical Alzheimer’s disease: Definition, natural history, and diagnostic criteria. Alzheimers Dement.

[9] Henriksen K, O’Bryant SE, Hampel H, Trojanowski JQ, Montine TJ, Jeromin A, Blennow K, Lönneborg A, Wyss-Coray T, Soares H, Bazenet C, Sjögren M, Hu W, Lovestone S, Karsdal MA, Weiner MW, for the Blood-Based Biomarker Interest Group The future of blood-based biomarkers for Alzheimer’s disease. Alzheimers Dement.

[10] Pan Y, Terpstra E, Wang Y, Qiao F, Wang J, Tong Y, Pan B Dysregulation and Diagnostic Potential of microRNA in Alzheimer’s Disease. J Alzheimers Dis.

[11] Wu HZ, Ong KL, Seeher K, J Armstrong N, Thalamuthu A, Brodaty H, Sachdev P, Mather K Circulating microRNAs as biomarkers of Alzheimer’s disease: A systematic review. J Alzheimers Dis.

[12] Li Y, Qui C, Tu J, Geng B, Yang J, Jiang T, Cui Q HMDD v2.0: a database for experimentally supported human microRNA and disease associations. Nucleic Acids Research.

[13] Koturbash I, Tolleson WH, Guo L, Yu D, Chen S, Hong H, Mattes W, Ning B microRNAs as pharmacogenomics biomarkers for drug efficacy and drug safety assessment. Biomark. Med.

[14] Mitchell PS, Parkin RK, Kroh EM, Fritz BR, Wyman SK, Pogosova-Agadjanyan EL, Peterson A, Noteboom J, O’Briant KC, Allen A, Lin DW, Urban N, Drescher CW Circulating microRNAs as stable blood-based markers for cancer detection. Proc Natl Acad Sci U S A.

[15] Fabbri M, Paone A, Calore F, Galli R, Gaudio E, Santhanam R, Lovat F, Fadda P, Mao C, Nuovo GJ, Zanesi N, Crawford M, Ozer GH, Wernicke D, Alder H, Caligiuri MA, Nana-Sinkam P, Perrotti D, Croce CM MicroRNAs bind to Toll-like receptors to induce prometastatic inflammatory response. Proc Natl Acad Sci U S A.

[16] Valadi H, Ekstrom K, Bossios A, Sjostrand M, Lee JJ, Lotvall JO Exosome-mediated transfer of mRNAs and microRNAs is a novel mechanism of genetic exchange between cells. Nat. Cell Biol.

[17] Pegtel DM, Cosmopoulos K, Thorley-Lawson DA, Van Eijndhoven MA, Hopmans ES, Lindenberg JL, de Gruijl TD, Würdinger T, Middeldorp JM Functional delivery of viral miRNAs via exosomes. Proc. Natl. Acad. Sci. U.S.A.

[18] Mittelbrunn M, Gutiérrez-Vázquez C, Villarroya-Beltri C, González S, Sánchez-Cabo F, MÁ González, Bernad A, Sánchez-Madrid F Unidirectional transfer of microRNA-loaded exosomes from T cells to antigen-presenting cells. Nat. Commun.

[19] Creemers EE, Tijsen AJ, Pinto YM Circulating microRNAs novel biomarkers and extracellular communicators in cardiovascular disease?. Circ Res.

[20] Geekiyanage H, Jicha GA, Nelson PT, Chan C Blood serum, miRNA: non-invasive biomarkers for Alzheimer’s disease. Experimental neurology.

[21] Kumar P, Dezso Z, MacKenzie C, Oestreicher J, Agoulnik S, Byrne M, Bernier F, Yanagimachi M, Aoshima K, Oda Y Circulating miRNA biomarkers for Alzheimer’s disease. PloS one.

[22] Leidinger P, Backes C, Deutscher S, Schmitt K, Mueller SC, Frese K, Haas J, Ruprecht K, Paul F, Stähler C, Lang CJ, Meder B, Bartfai T, Meese E, Keller A A blood based 12-miRNA signature of Alzheimer disease patients. Genome Biol.

[23] Keller A, Backes C, Haas J, Leidinger P, Maetzler W, Deuschle C, Berg D, Ruschil C, Galata V, Ruprecht K, Stähler C, Würstle M, Sickert D, Gogol M, Meder B, Meese E Validating Alzheimer’s disease micro RNAs using next-generation sequencing. Alzheimers Dement.

[24] Tan L, Yu JT, Tan MS, Liu QY, Wang HF, Zhang W, Jiang T, Tan L Genome-wide serum microRNA expression profiling identifies serum biomarkers for Alzheimer’s disease. J Alzheimers Dis.

[25] Tan L, Yu JT, Liu QY, Tan MS, Zhang W, Hu N, Wang YL, Sun L, Jiang T, Tan L Circulating miR-125b as a biomarker of Alzheimer’s disease. Journal of the neurological sciences.

[26] Liu CG, Song J, Zhang YQ, Wang PC MicroRNA-193b is a regulator of amyloid precursor protein in the blood and cerebrospinal fluid derived exosomal microRNA-193b is a biomarker of Alzheimer’s disease. Molecular medicine reports.

[27] Burgos K, Malenica I, Metpally R, Courtright A, Rakela B, Beach T, Shill H, Adler C, Sabbagh M, Villa S, Tembe W, Craig D, Van Keuren-Jensen K Profiles of extracellular miRNA in cerebrospinal fluid and serum from patients with Alzheimer’s and Parkinson’s diseases correlate with disease status and features of pathology. PLoS One.

[28] Satoh JI, Kino Y, Niida S MicroRNA-Seq Data Analysis Pipeline to Identify Blood Biomarkers for Alzheimer’s Disease from Public Data. Biomarker insights.

[29] Lugli G, Cohen AM, Bennett DA, Shah RC, Fields CJ, Hernandez AG, Smalheiser NR Plasma Exosomal miRNAs in Persons with and without Alzheimer Disease: Altered Expression and Prospects for Biomarkers. PloS one.

[30] Schipper HM, Maes OC, Chertkow HM, Wang E MicroRNA expression in Alzheimer blood mononuclear cells. Gene Regul Syst Bio.

[31] Bekris LM, Lutz F, Montine TJ, Yu CE, Tsuang D, Peskind ER, Leverenz JB MicroRNA in Alzheimer’s disease: an exploratory study in brain, cerebrospinal fluid and plasma. Biomarkers.

[32] Liu CG, Wang JL, Li L, Wang PC MicroRNA-384 regulates both amyloid precursor protein and β-secretase expression and is a potential biomarker for Alzheimer’s disease. Int J Mol Med.

[33] Liu CG, Wang JL, Li L, Xue LX, Zhang YQ, Wang PC MicroRNA-135a and-200b, potential Biomarkers for Alzheimer's disease, regulate β secretase and amyloid precursor protein. Brain res.

[34] Kiko T, Nakagawa K, Tsuduki T, Furukawa K, Arai H, Miyazawa T MicroRNAs in plasma and cerebrospinal fluid as potential markers for Alzheimer’s disease. J Alzheimers Dis.

[35] Bhatnagar S, Chertkow H, Schipper HM, Yuan Z, Shetty V, Jenkins S, Jones T, Wang E Increased microRNA-34c abundance in Alzheimer’s disease circulating blood plasma. Front Mol Neurosci.

[36] Galimberti D, Villa C, Fenoglio C, Serpente M, Ghezzi L, Cioffi SM, Arighi A, Fumagalli G, Scarpini E Circulating miRNAs as potential biomarkers in Alzheimer’s disease. J Alzheimers Dis.

[37] Ren RJ, Zhang YF, Dammer EB, Zhou Y, Wang LL, Liu XH, Feng BL, Jiang GX, Chen SD, Wang G, Cheng Q (2015). Peripheral Blood MicroRNA Expression Profiles in Alzheimer’s Disease: Screening, Validation, Association with Clinical Phenotype and Implications for Molecular Mechanism. Mol Neurobiol.

[38] Sørensen SS, Nygaard AB, Christensen T miRNA expression profiles in cerebrospinal fluid and blood of patients with Alzheimer’s disease and other types of dementia - an exploratory study. Transl Neurodegener.

[39] Guedes JR, Santana I, Cunha C, Duro D, Almeida MR, Cardoso AM, Pedroso de Lima MC, Cardoso AL MicroRNA deregulation and chemotaxis and phagocytosis impairment in Alzheimer’s disease. Alzheimers Dement (Amst).

[40] Jia LH, Liu YN Downregulated serum miR-223 servers as biomarker in Alzheimer’s disease. Cell Biochem Funct.

[41] Cheng L, Doecke JD, Sharples RA, Villemagne VL, Fowler CJ, Rembach A, Martins RN, Rowe CC, Macaulay SL, Masters CL, AF; Hill Australian Imaging, Biomarkers and Lifestyle (AIBL) Research Group. Prognostic serum miRNA biomarkers associated with Alzheimer’s disease shows concordance with neuropsychological and neuroimaging assessment. Mol Psychiatry.

[42] Zhu Y, Li C, Sun A, Wang Y, Zhou S (2015). Quantification of microRNA-210 in the cerebrospinal fluid and serum: Implications for Alzheimer’s disease. Exp Ther Med.

[43] Dong H, Li J, Huang L, Chen X, Li D, Wang T, Hu C, Xu J, Zhang C, Zen K, Xiao S Serum MicroRNA Profiles Serve as Novel Biomarkers for the Diagnosis of Alzheimer’s Disease. Disease Markers.

[44] Villa C, Fenoglio C, De Riz M, Clerici F, Marcone A, Benussi L, Ghidoni R, Gallone S, Cortini F, Serpente M, Cantoni C, Fumagalli G, Martinelli Boneschi F Role of hnRNP-A1 and miR-590-3p in neuronal death: genetics and expression analysis in patients with Alzheimer disease and frontotemporal lobar degeneration. Rejuvenation Res.

[45] Villa C, Ridolfi E, Fenoglio C, Ghezzi L, Vimercati R, Clerici F, Marcone A, Gallone S, Serpente M, Cantoni C, Bonsi R, Cioffi S, Cappa S Expression of the transcription factor Sp1 and its regulatory hsa-miR-29b in peripheral blood mononuclear cells from patients with Alzheimer’s disease. J Alzheimers Dis.

[46] Wang T, Chen K, Li H, Dong S, Su N, Liu Y, Cheng Y, Dai J, Yang C, Xiao S The feasibility of utilizing plasma miRNA107 and BACE1 messenger RNA gene expression for clinical diagnosis of amnestic mild cognitive impairment. J Clin Psychiatry.

[47] Reijs BL, CE2 Teunissen, Goncharenko N, Betsou F, Blennow K, Baldeiras I, Brosseron F, Cavedo E, Fladby T, Froelich L, Gabryelewicz T, Gurvit H, Kapaki E The Central Biobank and Virtual Biobank of BIOMARKAPD: A Resource for Studies on Neurodegenerative Diseases. Front Neurol.

[48] O’Bryant SE, Gupta V, Henriksen K, Edwards M, Jeromine A, Lista S, Bazenet C, Soares H, Lovestone S, Hampel H, Montine T, Blennow K, Foroud T Guidelines for the standardization of preanalytic variables for blood-based biomarker studies in Alzheimer’s disease research. Alzheimers Dement.

[49] Blondal T, Nielsen SJ, Baker A, Andreasen D, Mouritzen P, Teilum MW, Dahlsveen IK Assessing sample and miRNA profile quality in serum and plasma or other biofluids. Methods.

[50] Mestdagh P, Van Vlierberghe P, De Weer A, Muth D, Westermann F, Speleman F, Vandesompele J A novel and universal method for microRNA RT-qPCR data normalization. Genome Biol.

[51] Andersen CL, Jensen JL, Orntoft TF Normalization of Real-Time Quantitative Reverse Transcription-PCR Data: A Model-Based Variance Estimation Approach to Identify Genes Suited for Normalization, Applied to Bladder and Colon Cancer Data Sets. Cancer Res.

[52] Robin X, Turck N, Hainard A, Tiberti N, Lisacek F, Sanchez JC, Müller M. pROC: an open-source package for R and S+ to analyze and compare ROC curves. BMC Bioinformatics.

[53] TargetScanHuman: Prediction of microRNA targets. http://www.targetscan.org/vert_71/.

[54] Chou CH, Chang NW, Shrestha S, Hsu SD, Lin YL, Lee WH, Yang CD, Hong HC, Wei TY, Tu SJ, Tsai TR, Ho SY, Jian TY miRTarBase 2016: updates to the experimentally validated miRNA-target interactions database. Nucleic Acids Research.

[55] KEGG: Kyoto Encyclopedia of Genes and Genomes.

[56] Luo W, Brouwer C Pathview: an R/Bioconductor package for pathway-based data integration and visualization. Bioinformatics.

[57] Lau P, Bossers K, Janky R, Salta E, Frigerio CS, Barbash S, Rothman R, Sierksma AS, Thathiah A, Greenberg D, Papadopoulou AS, Achsel T, Ayoubi T Alteration of the microRNA network during the progression of Alzheimer’s disease. EMBO Mol Med.

[58] Wojsiat J, Prandelli C, Laskowska-Kaszub K, Martín-Requero A, Wojda U Oxidative stress and aberrant cell cycle in Alzheimer’s disease lymphocytes: diagnostic prospects. J Alzheimers Dis.

[59] Schnöder L, Hao W, Qin Y, Liu S, Tomic I, Liu X, Fassbender K, Liu Y Deficiency of Neuronal p38α MAPK Attenuates Amyloid Pathology in Alzheimer Disease Mouse and Cell Models through Facilitating Lysosomal Degradation of BACE1. Journal of Biological Chemistry. J Biol Chem.

[60] Barone E, Butterfield DA Insulin resistance in Alzheimer disease: Is heme oxygenase-1 an Achille’s heel?. Neurobiol Dis.

[61] Rani V, Deshmukh R, Jaswal P, Kumar P, Bariwal J Alzheimer’s disease: Is this a brain specific diabetic condition?. Physiol Behav.

[62] Lanni C, Racchi M, Memo M, Govoni S, Uberti D p53 at the crossroads between cancer and neurodegeneration. Free Radic Biol Med.

[63] Kudo W, Lee HP, Smith MA, Zhu X, Matsuyama S, Lee HG Inhibition of Bax protects neuronal cells from oligomeric Aβ neurotoxicity. Cell death & disease.

[64] Perluigi M, Barone E, Di Domenico F, Butterfield DA Aberrant protein phosphorylation in alzheimer disease brain disturbs pro-survival and cell death pathways. Biochim Biophys Acta.

[65] Kim S, Sato Y, Mohan PS, Peterhoff C, Pensalfini A, Rigoglioso A, Jiang Y, Nixon RA Evidence that the Rab5 effector appl1 mediates app-βctf-induced dysfunction of endosomes in Down syndrome and Alzheimer’s disease. Mol Psychiatry.

[66] Qi H, Prabakaran S, Cantrelle FX, Chambraud B, Gunawardena J, Lippens G, Landrieu I Characterization of Neuronal Tau Protein as a Target of Extracellular Signal-regulated Kinase. Journal of Biological Chemistry.

[67] Keller A, Meese E Can circulating miRNAs live up to the promise of being minimal invasive biomarkers in clinical settings?. Wiley Interdiscip Rev RNA.

[68] Sheinerman KS, Tsivinsky VG, Crawford F, Mullan MJ, Abdullah L, Umansky SR (2012). Plasma microRNA biomarkers for detection of mild cognitive impairment. Aging (Albany NY).

[69] Sheinerman KS, Tsivinsky VG, Abdullah L, Crawford F, Umansky SR (2013). Plasma microRNA biomarkers for detection of mild cognitive impairment: biomarker validation study. Aging (Albany NY).

[70] Xie B, Zhou H, Zhang R, Song M, Yu L, Wang L, Liu Z, Zhang Q, Cui D, Wang X, Xu S Serum miR-206 and miR-132 as potential circulating biomarkers for mild cognitive impairment. J Alzheimers Dis.

[71] Tominaga N, Kosaka N, Ono M, Katsuda T, Yoshioka Y, Tamura K, Lötvall J, Nakagama H, Ochiya T Brain metastatic cancer cells release microRNA-181c-containing extracellular vesicles capable of destructing blood-brain barrier. Nature communications.

[72] Shi M, Liu C, Cook TJ, Bullock KM, Zhao Y, Ginghina C, Li Y, Aro P, Dator R, He C, Hipp MJ Plasma exosomal α-synuclein is likely CNS-derived and increased in Parkinson’s disease. Acta Neuropathol.

[73] Kumar S, Reddy PH Are circulating microRNAs peripheral biomarkers for Alzheimer’s disease?. Biochim Biophys Acta.

[74] Bialopiotrowicz E, Kuzniewska B, Kachamakova-Trojanowska N, Barcikowska M, Kuznicki J, Wojda U Cell cycle regulation distinguishes lymphocytes from sporadic and familial AD patients. Neurobiol Aging.

[75] Esteras N, Alquézar C, Bermejo-Pareja F, Bialopiotrowicz E, Wojda U Martín-Requero. Downregulation of extracellular signal-regulated kinase 1/2 activity by calmodulin KII modulates p21Cip1 levels and survival of immortalized lymphocytes from Alzheimer’s disease patients. Neurobiol Aging.

[76] Buizza L, Cenini G, Lanni C, Ferrari-Toninelli G, Prandelli C, Govoni S, Buoso E, Racchi M, Barcikowska M, Styczynska M, Szybinska A, Butterfield DA, Memo M, Uberti D Conformational altered p53 as an early marker of oxidative stress in Alzheimer’s disease. PLoS One.

[77] Xiao Y, Yan W, Lu L, Wang Y, Lu W, Cao Y, Cai W p38/p53/miR-200a-3p feedback loop promotes oxidative stress-mediated liver cell death. Cell Cycle.

[78] Wang X, Jiang F, Song H, Li X, Xian J, Gu X MicroRNA-200a-3p suppresses tumor proliferation and induces apoptosis by targeting SPAG9 in renal cell carcinoma. Biochem Biophys Res Commun.

[79] Rechsteiner T, Schwarz E, Brock M, Huber L, Kohler M The micro-RNA hsa-miR-502-3p is down-regulated in the plasma of obstructive sleep apnea patients after 2 weeks of continuous positive airway pressure therapy withdrawal: Data from a randomized controlled trial. European Respiratory Journal.

[80] Daulatzai MA (2016). Cerebral hypoperfusion and glucose hypometabolism: Key pathophysiological modulators promote neurodegeneration, cognitive impairment, and Alzheimer’s disease. J Neurosci Res.

[81] Jin H, Yu M, Lin Y, Hou B, Wu Z, Li Z, Sun J MiR-502-3P suppresses cell proliferation, migration, and invasion in hepatocellular carcinoma by targeting SET. Onco Targets Ther.

[82] Ruszkiewicz J, Albrecht J Changes in the mitochondrial antioxidant systems in neurodegenerative diseases and acute brain disorders. Neurochem Int.

[83] Butzlaff M, Hannan SB, Karsten P, Lenz S, Ng J, Voßfeldt H, Prüßing K, Pflanz R, Schulz JB, Rasse T, Voigt A Impaired retrograde transport by the Dynein/Dynactin complex contributes to Tau-induced toxicity. Hum Mol Genet.

[84] Yılmaz ŞG, Erdal ME, Özge AA, Sungur MA Can Peripheral MicroRNA Expression Data Serve as Epigenomic (Upstream) Biomarkers of Alzheimer’s Disease?. OMICS.

[85] Zhang Y, Xing H, Guo S, Zheng Z, Wang H, Xu D MicroRNA-135b has a neuroprotective role via targeting of β-site APP-cleaving enzyme 1. Exp Ther Med.

[86] Yang G, Song Y, Zhou X, Deng Y, Liu T, Weng G, Yu D, Pan S MicroRNA-29c targets β-site amyloid precursor protein-cleaving enzyme 1 and has a neuroprotective role in vitro and in vivo. Mol Med Rep.

[87] Cosín-Tomás M, Antonell A, Lladó A, Alcolea D, Fortea J, Ezquerra M, Lleó A, Martí MJ, Pallàs M, Sanchez-Valle R, Molinuevo JL (2016). Plasma miR-34a-5p and miR-545-3p as Early Biomarkers of Alzheimer’s Disease: Potential and Limitations. Mol Neurobiol.

[88] Mandecka M, Budziszewska M, Barczak A, Pepłońska B, Chodakowska-Żebrowska M, Filipek-Gliszczyńska A, Nesteruk M, Styczyńska M, Barcikowska M, Gabryelewicz T (2016). Association between Cerebrospinal Fluid Biomarkers for Alzheimer’s Disease, APOE Genotypes and Auditory Verbal Learning Task in Subjective Cognitive Decline, Mild Cognitive Impairment, and Alzheimer’s Disease. J Alzheimers Dis.

