# Supplementary material for: Profile of 6 microRNA in blood plasma distinguish early stage Alzheimer’s disease patients from non-demented subjects
Source: Oncotarget. 2017 Feb 5;8(10):16122–43. doi: 10.18632/oncotarget.15109 (PMC5369952; doi:10.18632/oncotarget.15109)
Supplement: Supplementary file 1 [file oncotarget-08-16122-s001.pdf]

Profile of 6 microRNA in blood plasma distinguish early stage Alzheimer's disease patients from non-demented subjects

Supplementary Material

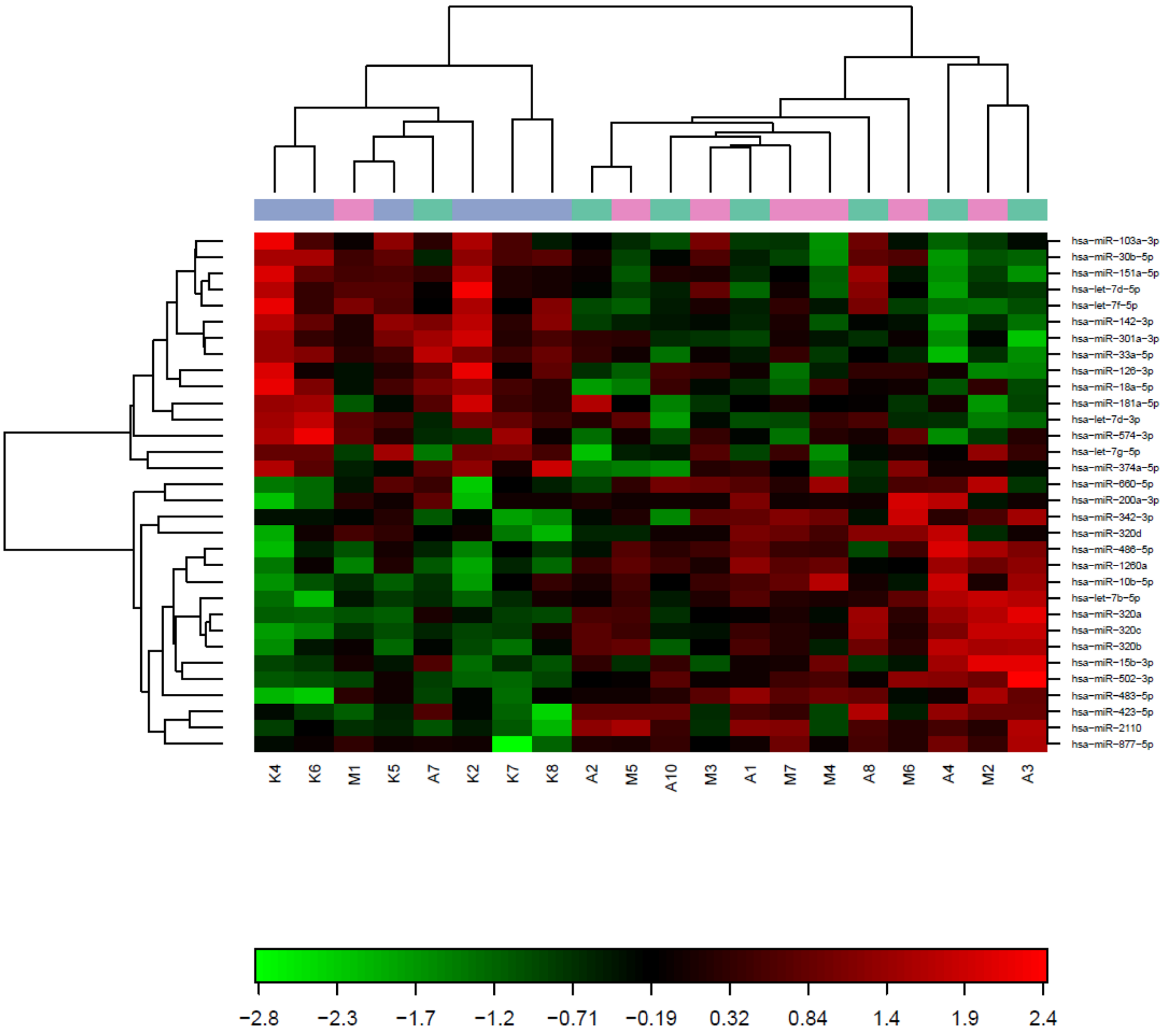

Supplementary Figure 1: shows top 32 differentially expressed miRNAs (one-way ANOVA, p value cut-off < 0.05) in the pilot experiment (Stage1). Unsupervised hierarchical clustering demonstrating the difference in global miRNAs pattern and segregation between samples from control non-demented subjects CTR1 versus both MCI-AD1 and AD1 groups. Each row represents one miRNA, and each column represents one sample (one subject). Panel above the heatmap represents groups: control subject (blue), MCI patients from the MCI-AD1 group (pink), AD patients from the AD1 group (green). IDs of samples are presented below the heatmap in the convention group id (K - control, M - MCI, A - AD) and sample number. Colors on the heatmap represents the Z-score: higher - red, lower - green.
