# Supplementary material for: Profile of 6 microRNA in blood plasma distinguish early stage Alzheimer’s disease patients from non-demented subjects
Source: Oncotarget. 2017 Feb 5;8(10):16122–43. doi: 10.18632/oncotarget.15109 (PMC5369952; doi:10.18632/oncotarget.15109)
Supplement: Supplementary file 2 [file oncotarget-08-16122-s002.docx]

Supplementary **Tab.1** shows sequences of the 15 plasma miRNAs differentiating MCI-AD (early AD) and AD patients from non-demented control subjects using Exiqon’s miRCURY LNA qRT-PCR panel (Stage 1). 9 novel and 6 previously reported AD biomarker candidates are shown.

| miRNA | Reference  database | Sequence |
| --- | --- | --- |
| Novel miRNA | | |
| hsa-miR-151a-5p | miRBase &Exiqon | UCGAGGAGCUCACAGUCUAGU |
| hsa-miR-30b-5p | miRBase &Exiqon | UGUAAACAUCCUACACUCAGCU |
| hsa-miR-486-5p | miRBase &Exiqon | UCCUGUACUGAGCUGCCCCGAG |
| hsa-miR-33a-5p | miRBase &Exiqon | GUGCAUUGUAGUUGCAUUGCA |
| hsa-miR-483-5p | miRBase &Exiqon | AAGACGGGAGGAAAGAAGGGAG |
| hsa-miR-18a-5p | miRBase &Exiqon | UAAGGUGCAUCUAGUGCAGAUAG |
| hsa-miR-320a | miRBase &Exiqon | AAAAGCUGGGUUGAGAGGGCGA |
| hsa-miR-320b | miRBase &Exiqon | AAAAGCUGGGUUGAGAGGGCAA |
| hsa-miR-320c | miRBase &Exiqon | AAAAGCUGGGUUGAGAGGGU |
| Reported miRNA | | |
| hsa-miR-502-3p | miRBase &Exiqon | AAUGCACCUGGGCAAGGAUUCA |
| hsa-miR-103a-3p | miRBase &Exiqon | AGCAGCAUUGUACAGGGCUAUGA |
| hsa-miR-301a-3p | miRBase &Exiqon | CAGUGCAAUAGUAUUGUCAAAGC |
| hsa-miR-142-3p | miRBase &Exiqon | UGUAGUGUUUCCUACUUUAUGGA |
| hsa-miR-200a-3p | miRBase &Exiqon | UAACACUGUCUGGUAACGAUGU |
| hsa-miR-1260a | miRBase &Exiqon | AUCCCACCUCUGCCACCA |

Supplementary **Tab.2** shows putative cellular effectors of 9 novel (A) and 6 previously reported (B) AD biomarker candidate miRNAs identified by searching the MirTarBase and KEGG’s neurodegenerative diseases and nervous system pathway database. All target proteins that had at least 2 or more hits in the databases are shown. Several example target genes for only one miRNAs are included.

A.

| Gene/pathway | Hits | Novel miRNAs |
| --- | --- | --- |
| MAPK | 6 | 483-5p, 30b-5p, 33a-5p, 18a-5p, 320b, 320a |
| Cx V | 5 | 483-5p, 151a-5p, 30b-5p, 320b, 320a |
| Cx I | 4 | 483-5p, 30b-5p, 320c, 320a |
| Cx IV | 4 | 18a-5p, 320b, 320a, 320c |
| Sema 7A | 4 | 33a-5p, 320b, 320a, 320c |
| p73 | 3 | 320b, 320a, 320c |
| Dynactin | 3 | 486-5p, 30b-5p, 18a-5p |
| CrK | 3 | 320b, 320a, 320c |
| ERK | 3 | 483-5p, 320b and 320a |
| RSK | 3 | 320b, 320a,320c |
| GLNT | 3 | 320b, 320a,320c |
| p53 | 2 | 30b-5p, 18a-5p |
| IGFIR | 2 | 486-5p, 320a |
| Bcl-2 | 2 | 30b-5p, 18a-5p |
| Rap 1 | 2 | 30b-5p, 18a-5p |
| JNK | 2 | 30b-5p, 33a-5p |
| Rab 5 | 2 | 33a-5p, 18a-5p |
| Ubc 6/7 | 2 | 18a-5p, 320a |
| Gs | 2 | 18a-5p, 320b |
| GLNS | 2 | 320b, 320a |
| mPTP | 2 | 320a,320c |
| BACE | 1 | 483-5p |
| PARKIN | 1 | 483-5p |
| SOD2 | 1 | 502-3p |
| p38 | 1 | 18a-5p |
| GAPD | 1 | 320a |
| CaM | 1 | 320a |
| NFkB | 1 | 320a |

B.

| Gene/pathway | Hits | Reported miRNAs |
| --- | --- | --- |
| Dynactin | 2 | 502-3p, 142-3p |
| CREB | 2 | 103a-3p, 142-3p |
| CaM | 2 | 301a-3p, 142-3p |
| PrP | 2 | 301a-3p, 1260a |
| FRS2 | 2 | 142-3p, 502-3p |
| GABR | 2 | 1260a, 103a-3p |
| PP2A | 2 | 142-3p, 103a-3p |
| CrK | 2 | 103a-3p, 502-3p |
| Kinesin | 2 | 142-3p, 103a-3p |
| MAPK | 2 | 301a-3p, 200a-3p |
| PI3K | 2 | 142-3p, 103a-3p |
| SSTR | 2 | 301a-3p, 502-3p |
